# Design principles of molecular networks revealed by global comparisons and composite motifs

**DOI:** 10.1186/gb-2006-7-7-r55

**Published:** 2006-07-19

**Authors:** Haiyuan Yu, Yu Xia, Valery Trifonov, Mark Gerstein

**Affiliations:** 1 Department of Molecular Biophysics and Biochemistry, Whitney Avenue, Yale University, New Haven, CT 06520, USA

## Abstract

**Background:**

Molecular networks are of current interest, particularly with the publication of many large-scale datasets. Previous analyses have focused on topologic structures of individual networks.

**Results:**

Here, we present a global comparison of four basic molecular networks: regulatory, co-expression, interaction, and metabolic. In terms of overall topologic correlation - whether nearby proteins in one network are close in another - we find that the four are quite similar. However, focusing on the occurrence of local features, we introduce the concept of composite hubs, namely hubs shared by more than one network. We find that the three 'action' networks (metabolic, co-expression, and interaction) share the same scaffolding of hubs, whereas the regulatory network uses distinctly different regulator hubs. Finally, we examine the inter-relationship between the regulatory network and the three action networks, focusing on three composite motifs - triangles, trusses, and bridges - involving different degrees of regulation of gene pairs. Our analysis shows that interaction and co-expression networks have short-range relationships, with directly interacting and co-expressed proteins sharing regulators. However, the metabolic network contains many long-distance relationships: far-away enzymes in a pathway often have time-delayed expression relationships, which are well coordinated by bridges connecting their regulators.

**Conclusion:**

We demonstrate how basic molecular networks are distinct yet connected and well coordinated. Many of our conclusions can be mapped onto structured social networks, providing intuitive comparisons. In particular, the long-distance regulation in metabolic networks agrees with its counterpart in social networks (namely, assembly lines). Conversely, the segregation of regulator hubs from other hubs diverges from social intuitions (as managers often are centers of interactions).

## Abstract 2

A global comparison of the four basic molecular networks in yeast - regulatory, co-expression, interaction and metabolic - reveals general design principles.

## Background

**Figure 1:**
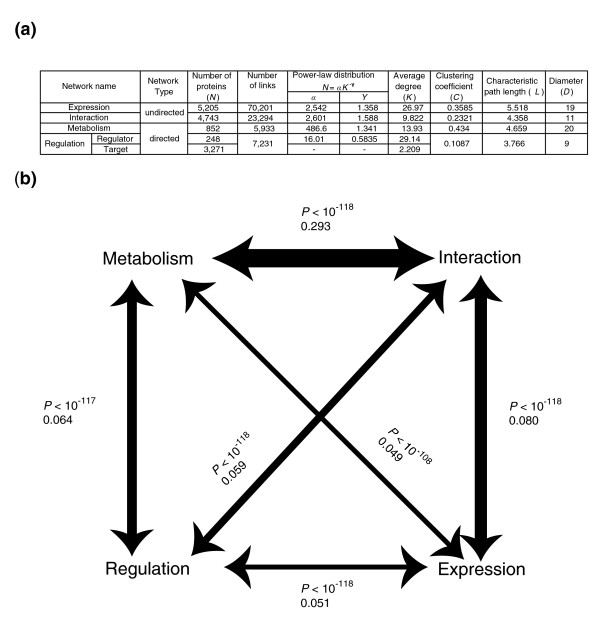
Global comparison of all four networks. (**a**) Topological statistics of all four networks. Because the degrees in the metabolic network are not divided into outward and inward degrees, we treated the metabolic network as an undirected network when calculating the average degree. (**b**) Association diagram between all four networks. The association between networks is measured by Cramer's *V*. The thickness of the line between two networks is proportional to the corresponding *V*. *P *values are calculated using standard χ^2 ^tests.

Traditionally, each protein has been studied individually as a fundamental functioning element within the cell. In the post-genomic era, however, proteins are often viewed and studied as interoperating components within larger cooperative networks [1]. Biological networks are topics of great current interest. With the publication of a number of large genome-wide expression, interaction, regulatory and metabolic datasets, especially in yeast [2-9], we can now construct four networks representing these four processes (see Materials and methods; Figure 1a).

### Importance of the four networks

We chose these four networks because they are the most commonly studied networks in yeast and because they can be easily related to the central dogma of molecular biology, which describes the basic (genetic) information flow in a cell. There are also other types of biological networks, such as synthetic lethal networks and chromosomal order networks [10,11]; however, these networks do not overlap with the central dogma and are, therefore, not the focus of this paper. Furthermore, most of these networks are not suitable for large-scale topological analysis because we do not have enough information on them.

Another important reason for us to choose these four networks is that there are many appealing analogies between these biological networks and corresponding social networks [12-14]. Because people have clear intuition for social networks, based on daily experiences, these analogies can make molecular networks easier to comprehend. For example, social hierarchy networks resemble the regulatory networks in that they define who has to obey orders from whom. Social acquaintance networks describe who is known to whom in the society and are, therefore, similar to interaction networks in biology [13,14]. Finally, enzymes at different steps of the metabolic network can be considered as workers at different steps of the assembly line in a factory.

### Composite features in combined networks

Individual networks have been globally characterized by a variety of graph-theoretic statistics (Additional data file 1), such as degree distribution, clustering coefficient (*C*), characteristic path length (*L*) and diameter (*D*) [12,15,16]. Barabási and Albert [12] proposed a 'scale-free' model in which most of the nodes have very few links, with only a few of them (hubs) being highly connected. In addition to topological statistics and hubs, network motifs provide another important summary of networks. These are over-represented sub-graph patterns in networks, and they are considered as basic building blocks of large-scale network structures [17]. Recently, Yeger-Lotem *et al*. [18] combined the interaction and regulatory networks in yeast and searched for patterns in the combined network.

Here, we build on previous network studies and extend them in novel directions by combining all four networks in our analysis. Our goal is to examine the topological features of our combined network. We call these 'composite features' to distinguish them from those in single networks (see Materials and methods). By analyzing these in all four networks, we were able to find some basic principles characterizing biological networks. For example, previous studies have shown most biological networks are scale-free, having only a few hubs as the most important and vulnerable points [12,15]. It is quite reasonable to assume that our four networks will share the same set of hubs as explained in detail below. However, we analyzed the composite hubs among the four networks and showed that the regulatory network tends to use a distinctly different set of hubs compared to the other three networks. Furthermore, one fundamental question in biology is how the cell uses transcription factors (TFs) to regulate and coordinate the expression of thousands of genes in response to internal and external stimuli [8,19-21]. Through examining composite motifs, we could potentially shed some light on this question. In particular, we show that the expression of enzymes at different steps of the same pathway tends to have time-delayed relationships mediated by inter-regulating TFs.

## Results and discussion

### Overall comparisons of all four networks

We calculated many topological statistics in all four networks, which are summarized in Figure 1a. All four networks display 'scale-free' and 'small-world' properties. However, the regulatory network is different from other networks in that its clustering coefficient is exceptionally small. This is because most of the target genes are not TFs. Therefore, the target genes of the same regulator tend not to inter-regulate one another. Moreover, since the regulatory network is directed, it is divided into regulator and target sub-networks when calculating the degree distribution. It has been shown that the regulator network is a scale-free network. But, the target network might have an exponential degree distribution, instead [22]. This means that there are no hubs in the target network. Therefore, when we examined the hubs and composite hubs in the regulatory network, we focused only on the regulator population. This also makes sense biologically, because we are more interested in how a gene's expression is regulated in different networks; the regulators (that is, TFs) are the ones that carry out the regulatory functions.

Furthermore, we analyzed the relationships between different networks. Since the relative position of nodes in a network is one of the most important features of the network, we examined the relationships between networks using their distance matrices, that is, distances between all protein pairs. We divided all pairs of proteins in a network into three groups: connected pairs; close pairs (distance = 2); and distant pairs (distance ≥3). We used Cramer's *V*, a measurement derived from χ^2 ^statistics, to examine the association between networks, that is, whether pairs of proteins in one group of a network tend to be in the same group of another network. Our calculations confirm that all networks are indeed significantly related to each other (Figure 1b). We also tried many other metrics of relatedness - for example, Pearson correlation coefficient, mutual information, contingency coefficient, and association score. They all show similar results (see Supplementary Table 1 in Additional data file 1).

### Composite hubs tend to be more essential than hubs in single networks

**Figure 2:**
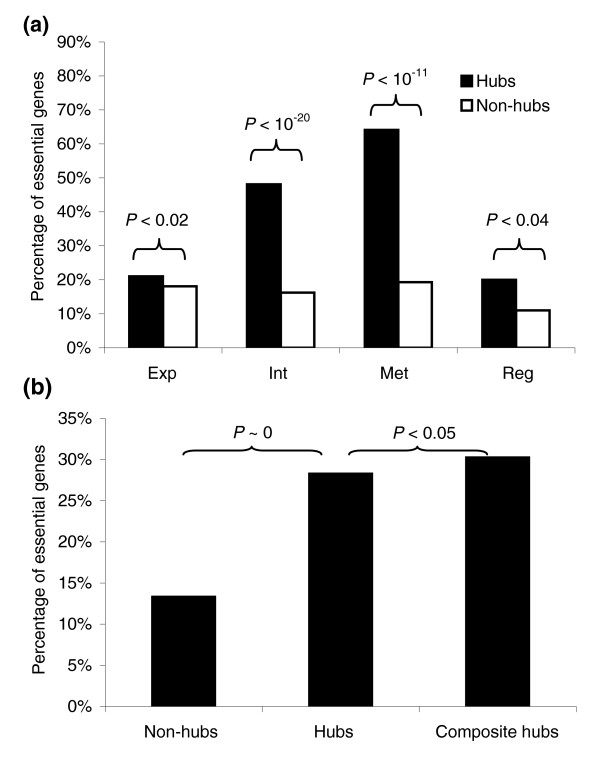
Analysis of the essentiality of hubs and composite hubs. (**a**) Comparison of the percentages of essential genes in hubs and non-hubs in different networks. *P *values measure the significance of differences between the percentages for hubs and non-hubs. (**b**) Comparison of the percentages of essential genes in non-hubs, hubs and composite hubs. In this figure, we excluded all composite hubs when calculating the percentage for hubs. Due to the limited number of tri-hubs, we combined them with bi-hubs. *P *values measure the significance of the differences between neighboring bars. Met, the metabolic network; Int, the interaction network; Exp, the co-expression network; and Reg, the regulatory network (in Figures 2 and 3, we only consider the regulator population in the regulatory network).

Previous studies have shown that hubs are the scaffolding of scale-free networks with great importance for their stability [12]. In particular, hubs in interaction networks tend to be essential [15], and they tend to be more conserved through evolution than non-hubs [23]. Therefore, we next examined the fraction of essential genes among hubs and non-hubs in different networks. Not surprisingly, hubs in all networks tend to be essential (Figure 2a; here we only consider the regulator population within the regulatory network). The results agree well with previous studies [15,24]. Furthermore, we analyzed the essentiality of composite hubs. Figure 2b clearly shows that, while hubs in single networks (that is, normal hubs) tend to be essential compared with non-hubs, composite hubs have an even higher tendency to be essential than normal hubs. Due to the essentiality of normal hubs, composite hubs should be more essential (Additional data file 1), which agrees well with our observation. Because of the limited statistics, we cannot determine whether there are additional reasons for the increased tendency of composite hubs to be essential (Supplementary Figure 1 in Additional data file 1).

**Figure 3:**
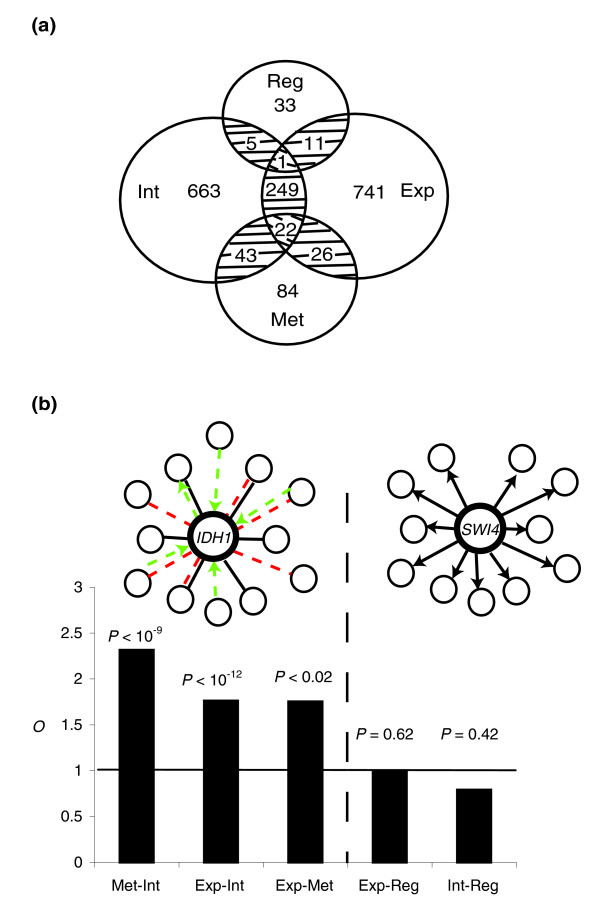
**Analysis of hub overlaps**. (**a**) Venn diagram describing hub overlaps between networks. Shaded areas represent composite hubs. (**b**) Fold enrichments of hub overlaps (*O*) between two networks relative to random expectation. The bars above the line (where *O *= 1) show that overlapping hubs between the two networks are more than expected. The schematic above the first three bars shows that action networks tend to share the same hubs. One of the tri-hubs is Idh1p, an isocitrate dehydrogenase involved in the tricarboxylic acid cycle connecting a number of different pathways [7]. It is also involved in a number of complexes, and is thus co-expressed with many other genes [5,6,40,49]. In this schematic, the solid circle represents the composite hub; open circles represent different proteins; black solid lines represent interaction relationships; red dashed lines represent co-expression relationships; green dashed arrows represent metabolic reactions. The schematic above the last two bars shows that the regulatory network uses a distinct set of hubs. For example, Swi4p is a major TF regulating the yeast cell cycle [50]. However, it is not a hub in any of the action networks. In this schematic, the solid circle represents the regulatory hub; open circles represent different proteins; black solid arrows represent regulatory relationships. *P *values measure the significance of the differences between the observed overlaps and the random expectation. The random expectation was calculated as described in Materials and methods. *P *values in this figure and all following figures were calculated using the cumulative binomial distribution (Additional data file 1). Met, the metabolic network; Int, the interaction network; Exp, the co-expression network; and Reg, the regulatory network (in Figures 2 and 3, we only consider the regulator population in the regulatory network).

In our analysis, composite hubs can be either bi-hubs (hubs in two of the four networks) or tri-hubs (hubs in three of the four networks). We identified hubs and composite hubs in all four networks (Figure 3a). Considering only the regulator population of the regulatory network, we were able to identify 334 bi-hubs and 23 tri-hubs. For example, *GCN4 *is a tri-hub involving interaction, co-expression, and regulatory networks. Gcn4p is a master regulator of amino acid biosynthetic genes in response to starvation and stress, with 111 known targets [25]. It is known to interact specifically with RNA polymerase II holoenzymes, Adap-Gcn5p co-activator complex, and many other proteins (16 in total) [26]. *GCN4 *was also co-expressed with 134 other genes in the cell-cycle experiments of Cho *et al*. [6]. No proteins are hubs in all four networks, because most enzymes are not TFs. Finally, we can show that the structure of biological networks in yeast is very different from the most obviously corresponding structures in social networks.

### Scaffolding of the regulatory network is different from other networks

Because all four biological networks are scale-free (Figure 1a; here we only consider the regulator population within the regulatory network), it can be shown that they should share the same hubs by chance alone due to hubs' essentiality (Additional data file 1). It is interesting to see whether this is indeed the case for biological networks, that is, whether they are built on the same scaffolding.

Our calculation shows that the scaffolding of three networks (metabolic, interaction and co-expression) tends to be the same, that is, hubs in one network tend to overlap with those in another when compared to random expectation (Figure 3b). The results agree with previous studies showing that interacting proteins tend to be co-expressed [27-30]. Furthermore, we calculated the random expectation by taking into consideration the fact that hubs tend to be essential [15,24]. We found that the hub overlap between networks could not be explained by simply considering the essentiality of hubs (Supplementary Figure 2 in Additional data file 1).

Surprisingly, hubs in the regulator network do not have the tendency to be hubs in other networks. Though counter-intuitive, this observation is reasonable in that most TFs and their targets do not tend to be co-expressed [31], and most TFs are unlikely to interact with their targets. Therefore, we divided the four networks into two classes: regulation and action. The action networks include the interaction, co-expression and metabolic networks. It is clear that the cell separates the regulatory network from the action networks. Since all action networks are governed by the regulatory network as discussed below, the separation potentially could provide stability to the cell (Supplementary Figure 5 in Additional data file 1).

Here we have excluded the comparison between regulator and metabolic networks because the two networks only share one common protein. It is possible to argue that our definition of hubs is somewhat arbitrary. But all results remain the same even when we used different cutoffs to define hubs. We further tested the functional composition of the overlapping proteins among networks, which is similar to that of each individual network and random expectation (Supplementary Figures 3 and 4 in Additional data file 1).

### Neighboring pairs in all action networks are co-regulated

Above, we separated the regulatory network from the others; now we show that the three action networks can be further subdivided into two groups (that is, short-range and long-range) based on how the genes in them are regulated by TFs. We investigated this through looking at composite motifs within the combined regulatory-action network. We focused on a few key motifs, which we call triangles, trusses, and bridges (see Materials and methods).

**Figure 4:**
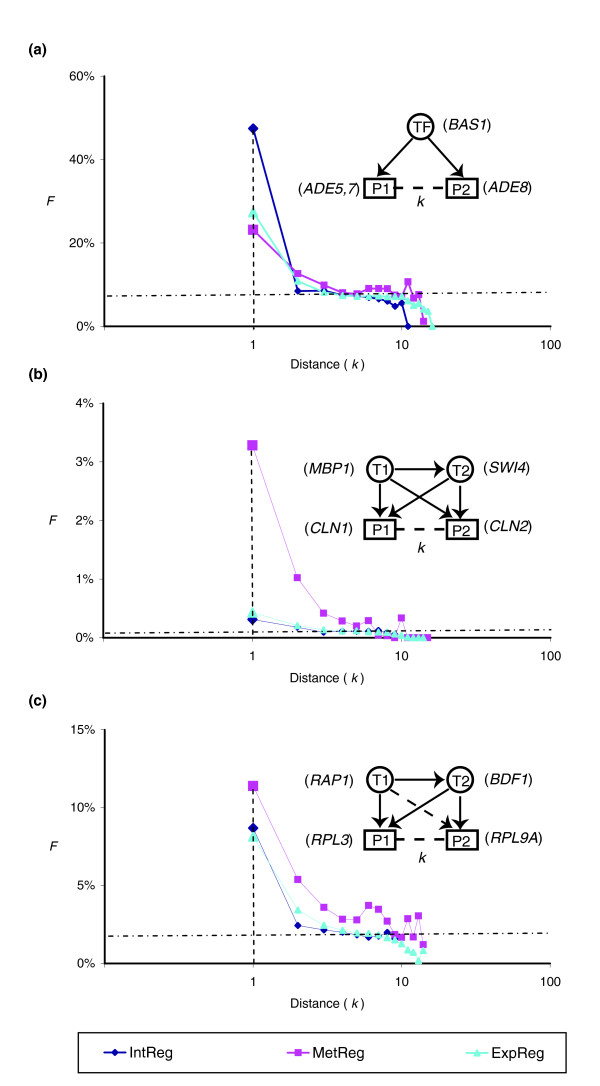
Fraction (*F*) of all P1-P2 pairs at distance *k *in a given combined network in a particular composite motif. Horizontal dashed lines indicate the random expectation. Vertical dashed lines indicate connected pairs in combined networks. (**a**) Triangles. The schematic shows that a triangle consists of three proteins: the common regulator TF regulates both P1 and P2. In all schematics, circles represent TFs, and rectangles represent non-TF genes. For example, ADE5, 7 and ADE8 are two subsequent enzymes in the purine biosynthesis pathway [7]. They are co-regulated by BAS1 [51]. (**b**) Trusses. The schematic shows that a truss consists of four proteins: T1 regulates T2, P1 and P2; T2 regulates P1 and P2. For example, Cln1p and Cln2p are two subunits of the *CDC28*-associated complex [4]. They are co-regulated by Mbp1p and Swi4p [52]. Mbp1p also regulates *SWI4 *[8,53]. (**c**) Semi-trusses. A semi-truss is an incomplete truss: either T2 does not regulate P1, or T1 does not regulate P2. For example, *RPL3 *and *RPL9A*, components of the ribosome large subunit, are co-expressed [6]. They are co-regulated by Bdf1p [54]. Rap1p regulates both *RPL3 *and *BDF1 *[8,55]. We also examined the occurrence of triangles and trusses between protein pairs connected in more than one network, termed highly combined networks. We only considered semi-trusses to get better statistics, since the number of full trusses in highly combined networks is too small to be used. In all highly combined networks, triangles and semi-trusses are enriched between protein pairs connected in more than one network (Figure 8 in Additional data file 1). Met, the metabolic network; Int, the interaction network; Exp, the co-expression network; and Reg, the regulatory network.

In a triangle, two genes (P1 and P2) are co-regulated by the same regulator (TF). Therefore, triangles should tend to occur between co-expressed gene pairs (Figure 4a). Since interacting proteins and co-enzymes are known to be co-expressed [20,30], we expected to see that triangles are enriched between the connected pairs in all three combined networks. Our results confirmed this expectation in that the percentage of triangles between connected pairs in all three networks are significantly higher than random, while the percentage between disconnected pairs is equal to or even lower than random (Figure 4a). In other words, connected pairs in all three networks tend to be co-regulated, which is in agreement with our expectation and with previous studies [20,30,31].

In a truss, two proteins share the same feed-forward loop (FFL; Figure 4b). FFLs are robust against noise [32]. Previous work has also shown that genes co-regulated by more than one regulator tend to be tightly co-expressed [31]. Therefore, trusses are designed to maintain stable co-expression between gene pairs. Their biological function is similar to that of triangles.

We examined the distributions of the enrichment of trusses in all three combined networks. As expected, the three distributions share similar patterns with that of triangles (Figures 4a,b). In all distributions, only connected pairs show enrichment of trusses, which further confirms the biological function of trusses. Given the fact that the regulatory network in yeast is far from complete, we believe that many actual trusses are missed by our analysis because some of the edges are missing in our dataset. To confirm this, we also looked at semi-trusses. A semi-truss is a truss with only one FFL (Figure 4c). We believe that many of these semi-trusses are actually full trusses given the incomplete nature of our dataset. Figure 4c shows highly similar results to those in Figure 4b, thus providing support for our conclusion.

Interestingly, it has been shown experimentally that triangles and trusses can also generate temporal programs of expression by having serial activation coefficients with different targets, which is quite intuitive and reasonable [33,34]. It should also be noted that some FFLs ('incoherent FFLs') could provide pulses and speeding responses, although the majority of FFLs are coherent, acting as 'persistence detectors' [35,36].

### Distant enzymes in the same pathway tend to have delayed expressions mediated by regulator bridges

**Figure 5:**
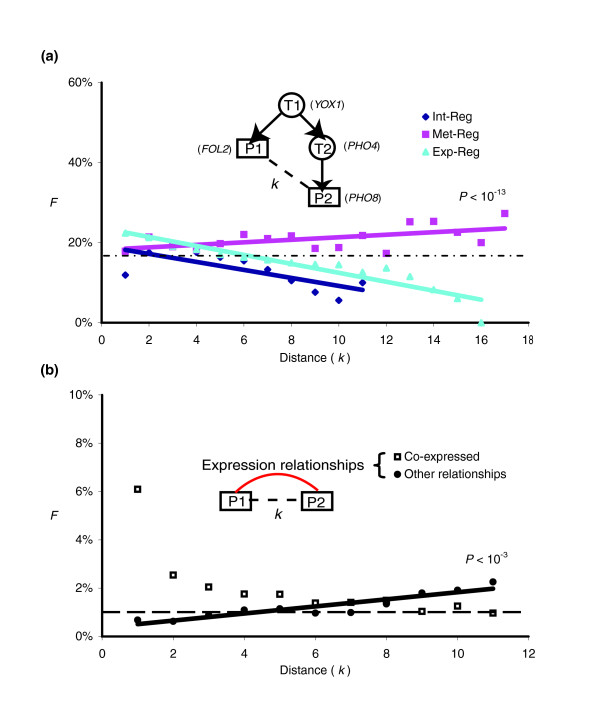
Fraction (*F*) of all P1-P2 pairs at distance *k *in a given combined network in a particular composite motif. Horizontal dashed lines indicate the random expectation. (**a**) Bridges. The schematic shows that a bridge consists of four proteins: T1 regulates T2 and P1; T2 regulates P2. For example, Fol2p and Pho8p are two subsequent enzymes involved in the folate biosynthesis pathway [7]. *FOL2 *is regulated by Yox1p [9]. *PHO8 *is regulated by Pho4p [56]. Yox1p also regulates *PHO4 *[9]. The *P *value in the figure indicates the significance of the different between the fraction of bridges between all disconnected enzyme pairs and the random expectation (Table 3 in Additional data file 1). The regression equation for Met-Reg: *F *= 0.003*k *+ 0.18; *R *= 0.56; *P *< 0.01. The regression equation for Int-Reg: *F *= -0.01*k *+ 0.19; *R *= 0.74; *P *< 10^-3^. The regression equation for Exp-Reg: *F *= -0.01*k *+ 0.24; *R *= 0.93; *P *< 10^-9^. *P *values here measure the significance of the correlation (R) in regression. (**b**) Composite motifs in the combined network of Met-Exp (that is co-expression motifs and shifted motifs). The schematic shows that composite motifs in Met-Exp consist of two proteins: P1 and P2. P1 and P2 have a distance of *k *in the metabolic network. They also have an expression relationship (co-expressed or others) in the co-expression network. The *P *value indicates that the fraction of protein pairs in shifted motifs in Met-Exp is significantly higher than expected. The regression equation for Met-Exp: F = 0.002*k *+ 0.0037; R = 0.92; P < 10^-8^. Met, the metabolic network; Int, the interaction network; Exp, the co-expression network; and Reg, the regulatory network.

In a bridge, protein P1 and regulator T2 are co-regulated by T1 and, thus, should be co-expressed. Only after the gene of T2 is expressed (transcribed) and translated can the protein product of T2 then bind to P2 and activate its expression. Therefore, the expressions of P1 and P2 should not be simultaneous, but rather have a time delay (Supplementary Figure 9 in Additional data file 1). We expected that bridges would tend to occur between gene pairs that are closely functionally related, but not necessarily co-expressed. We calculated the distributions of the occurrence of bridges between gene pairs with different distances in all three combined networks, (Figure 5a). The results are rather surprising, since, in interaction and co-expression networks, the tendency of forming bridges between protein pairs decreases as their distance increases. However, the tendency of forming bridges remains the same for enzymes with different distances in the same metabolic pathways. The tendency stays significantly higher than random even for far-away pairs (Supplementary Table 3 in Additional data file 1). Clearly, genes in the interaction and co-expression networks only have short-range regulatory relationships, whereas genes in the metabolic networks have long-range ones. (Another unlikely but possible hypothesis for this result is that there is a subtle bias in the metabolic network since it was mapped mostly based on small-scale experiments, unlike interaction and co-expression networks.)

We then analyzed the composite motifs in the combined metabolism-co-expression network. Figure 5b shows that co-enzymes tend to be co-expressed, and the tendency of co-expression decreases as the distance between the enzymes increases. On the other hand, enzymes in different steps of the same pathway tend to have expression relationships other than co-expression, typically time-delayed relationships (Supplementary Figure 7c in Additional data file 1). This tendency increases as the distance increases. The likelihood for far-away enzymes in the same pathway to have other expression relationships is significantly higher than random expectation. This observation shows that enzymes in the same pathway are not necessarily co-expressed; nevertheless, their expression needs to be well-coordinated for the whole pathway to function normally. This is the reason why bridges are enriched in disconnected enzyme pairs in the metabolic network (Figure 5a). Similar results were also found in other time-course expression experiments [37], but not in the interaction network (Additional data file 1). This conclusion is further supported by a specific case study in *Escherichia coli *amino acid biosynthesis pathways [33]. As we mentioned above, metabolic pathways in the cell are very similar to assembly lines in a factory. It is reasonable to assume that, without decreasing the efficiency of the whole assembly line, workers at downstream steps of the line do not have to show up for work until those at upstream steps have finished their job. Similarly, in terms of metabolic pathways, we observed that enzymes at downstream steps tend to be expressed after those at earlier steps. The bridge motifs are designed to manage such expression relationships between enzymes, and, therefore, to maintain normally functioning metabolic pathways in the cell.

## Conclusion

Here we examine the four most commonly studied networks in yeast. Previous work has shown that social networks share common characteristics with biological networks [12-14]. Our results further confirm this. In particular, many common social networks are related. We also found that biological networks, even though seemingly quite different, are clearly related to each other. In social networks, people under the same supervisor normally know each other, and, as such, may be said to be connected in acquaintance networks. Accordingly, in the biological networks, we observed that connected pairs in action networks tend to be co-regulated. More interestingly, distant enzymes in the same pathway show a surprising tendency to have delayed expression coordinated by regulator bridges. Although this phenomenon is readily understandable through an analogy to assembly lines, it is still striking to see it so strongly manifest in real biological networks. However, the structure of biological networks obviously has some differences from that of social networks. In a normal social context, it is reasonable to assume that a supervisor knows his or her staff. Therefore, supervisors with large staffs (that is, hubs in the social hierarchy) tend to be hubs in acquaintance networks. This is not the case for biological networks: the regulatory network uses a different set of hubs than the action networks.

Recently, Mazurie *et al*. [38] also analyzed the composite network motifs in the combined regulatory and interaction network. They used a similar approach to Yeger-Lotem *et al*. [18] and examined the composite motifs that are over-represented in a strictly mathematical sense. However, they found that the overabundance of these network motifs "does not have any immediate functional or evolutionary counterpart" [38]. These findings confirm that we should not only look at the most mathematically over-represented motifs, but that we should also focus on key, obviously functionally relevant ones, further highlighting the importance of our approach. In our analysis, we first identified composite motifs that could potentially have biological functions and examined the enrichment of these motifs in the combined network. Our results have clearly shown that the enrichment of some composite motifs is closely related with their function. For example, bridges are only enriched between far-away enzymes in the same pathway because the expression of these enzymes needs to be well coordinated.

## Materials and methods

### Biological networks

The regulatory network was created by combining five different datasets [8,9,22,31,39,40]. A link in the network is defined as a TF-target pair. We excluded DNA-binding enzymes (for example, PolIII) and general TFs (for example, TATA-box-binding protein) from the regulatory network.

The co-expression network was created using the microarray dataset of Cho *et al*. [6]. A link here is defined as a co-expressed gene pair with a correlation coefficient larger than or equal to 0.8. It is possible to argue that the cutoff (0.8) here is somewhat arbitrary. We repeated all relevant calculations using different cutoffs ranging from 0.5 to 0.9. All results remained the same (Additional data file 1).

The interaction network was created by combining various databases and large-scale experiments [2-5,41-43]. Because large-scale experiments are known to be error-prone [44], we only considered high-confidence protein pairs as true interacting pairs (likelihood ratios ≥300, *P *value < 10^-200 ^as estimated by the hypergeometric distribution; likelihood ratios measure the enrichment of interacting protein pairs with certain genomic features [45]; see Additional data file 1 for a detailed discussion).

The metabolic network was downloaded from the KEGG database [7]. However, the metabolic network is different from the other networks in that the nodes in the network are small molecules and they are connected by the enzymatic steps between them. To compare the metabolic network to others, we transformed the network in the following way: each enzyme was considered a node in the network, and enzymes working on adjacent steps were considered 'connected'. Whenever there is more than one enzyme in the same enzymatic step (that is, co-enzymes), we also consider all co-enzymes as 'connected'. Only main substrates and products were used to perform the transformation. Most co-factors and carriers (for example, ATP and H_2_O) were removed from all reactions.

All four networks are available through our supplementary website [46].

### Composite topological features

#### Composite hubs

We define hubs in a single network as the top 20% of the nodes with the highest degrees [19,24]. Accordingly, composite hubs are defined as the nodes that are hubs in more than one network.

#### Composite motifs

Yeger-Lotem *et al*. [18] defined composite motifs operationally as over-represented patterns in the combined network as compared to a randomized control. Using this criterion, they exhaustively searched through the combined network and were able to detect 1 two-node, 5 three-node and 63 four-node composite motifs. A similar study has also been performed by Zhang *et al*. [47]. Instead of automated detection of new composite motifs, we manually selected five basic composite motifs for further analysis because, as discussed below, these composite motifs summarize the most basic biological relationships between protein pairs within the four networks. Our analysis covered all four biological networks. We analyzed not only nearest neighbors, but also protein pairs that are further apart in each network. Most importantly, we were able to gain significant insights into the biological functions of the five composite motifs by comparing their patterns of occurrence in the combined networks.

### Definition of five composite motifs

We first examined the regulatory relationships between protein pairs in action networks and created three combined networks by combining the regulatory network with each of the other three networks. We defined three biologically meaningful composite motifs in all three combined networks, based on the fact that co-regulation (that is, that two proteins share the same regulator) and inter-regulation (that is, that the regulator of one protein regulates the regulator of another protein) are the two most basic regulatory relationships between a pair of proteins. The three basic composite motifs that we defined are: co-regulation motifs (triangles); integrated FFLs (trusses); and bridging motifs (bridges) (Supplementary Figure 6 in Additional data file 1). Yeger-Letem *et al*. [18] determined that triangles and trusses are significantly overrepresented motifs, but bridges are not. However, we are able to show the biological importance of bridges in the main discussion (see above).

We also created another combined network by combining the co-expression and metabolic networks. Qian *et al*. [48] developed a local clustering method to detect four expression relationships between gene pairs: co-expressed, time-shifted, inverted, and inverted time-shifted. Using the local clustering method, we defined two composite motifs in this combined network (Supplementary Figure 7 in Additional data file 1): the co-expression motif, a pair of enzymes at distance *k *in the metabolic network that are co-expressed; and the shifted motif, a pair of enzymes at distance *k *in the metabolic network that have expression relationships other than co-expression. Most of these pairs have time-shifted relationships.

For each of the above composite motifs, we determined its degree of enrichment at different distances in different action networks in the following way. We first counted the number of protein pairs at a certain distance *k *in each of the three action networks. Then, we calculated the fraction of pairs that are within a certain composite motif.

### Calculations of the random expectation of hub overlaps

To calculate random expectation of hub overlaps, we first created randomized networks for each biological network by randomly shuffling node degrees among proteins throughout the whole network. In this manner, the degree distributions of the original networks are conserved in randomized networks. Then, we calculated the overlap of hubs between the randomized networks of the two original networks. The procedure was repeated 1,000 times. The average overlap is considered as the random expectation.

An observed enrichment in hub overlap can be partly explained by the fact that hubs tend to be essential. In order to take into consideration hub essentiality, we created randomized networks by shuffling degrees only among genes that are either essential or non-essential. In this manner, the tendency for hubs to be essential is conserved in randomized networks. Other steps are the same as above.

Similarly, an observed enrichment in essentiality of composite-hubs compared to hubs in a single network can be at least partly explained by the fact that hubs generally tend to be essential. To prove this, we again created randomized networks where the tendency for hubs to be essential is conserved. We then compared observed essentiality enrichment in composite-hubs with calculations based on the randomized networks.

## Additional data files

The following additional data are available with the online version of this paper. Additional data file 1 is a PDF file containing the supplementary materials to the main manuscript, in which we introduce the details of many calculations performed in the main text and discuss many additional results supporting the conclusions in the main text.

## Supplementary Material

Additional data file 1 Supplementary figures and tables that introduce details of many calculations performed in the main text, and discussion of many additional results supporting the conclusions in the main text. Click here for file
