# Supplementary material for: Design principles of molecular networks revealed by global comparisons and composite motifs
Source: Genome Biol. 2006 Jul 19;7(7):R55. doi: 10.1186/gb-2006-7-7-r55 (PMC1779570; doi:10.1186/gb-2006-7-7-r55)
Supplement: Additional data file 1 — Supplementary figures and tables that introduce details of many calculations performed in the main text, and discussion of many additional results supporting the conclusions in the main text. [file gb-2006-7-7-r55-S1.pdf]

Supplementary materials

Topological statistics of networks (excerpt from [14])

The topological analysis of the networks provides quantitative insight into their basic organization. Four topological statistics of particular interest in network analysis are:

(1) Average degree (K). The degree of a node is the number of links that this node has with other nodes. The average degree of the whole network is the average of the degrees of all its individual nodes.

(2) Clustering coefficient (C). This is defined as the ratio of the number of existing links between a node's neighbors and the maximum possible number of links between them (similar to an odds ratio). The clustering coefficient of a network is the average of all its individual coefficients. This statistic can be used to determine the completeness of the network.

(3) Characteristic path length (L). The graph theoretical distance between two nodes is the minimum number of edges that is necessary to traverse from one node to the other. The characteristic path length of a network is the average of these minimum distances. It gives a measure of how close nodes are connected within the network.

(4) Diameter (D). The diameter of a network is the longest graph theoretical distance between any two nodes in the graph. Table 1c explains, in detail, the formulas that are used to calculate these statistics.

Until recently, classical random network theory was used to model complex networks. This was introduced by Erdös and Rényi. It assumes that any two nodes in the network are connected with random probability p and the degrees of the nodes follow a Poisson distribution, which has a strong peak at the average degree, K. Most random networks are highly homogenous, in that most nodes have the same number of links (degree), $k_i \approx K$, where k_i is the degree of the ith node. The chance of having nodes with k links falls off exponentially for large k (i.e. $P(k) \approx e^{-k}$), meaning that it is *very* unlikely that there will be any nodes of degree significantly larger than average.

To explain the heterogeneous nature of complex networks, Barabási and colleagues recently proposed a "scale-free" model in which the degree distribution in many large networks follows a power-law ($P(k) \approx k^{-r}$). A remarkable point about this distribution is that most of the nodes within these networks have very few links, with only a few of them (hubs) being highly connected. Many aspects of genomic biology have such a scale-free structure. Concurrently, Watts & Strogatz found that many networks can also be described as having a "small-world" property, i.e. they are defined as being both highly clustered and containing small characteristic path lengths (large C and small L).

Calculation of likelihood ratios

Jansen et al created a set of gold standard positives (truly interacting proteins) and a set of gold standard negatives (truly non-interacting proteins) for the prediction of interactions [43]. The likelihood ratio L for a certain feature f (e.g. two genes being co-regulated) is then defined as

$$L(f) = \frac{\frac{TP}{FP}}{\frac{P}{N}}$$

where P represents the total number of gold standard positives. N represents the total number of gold standard negatives. TP is the number of true positives (i.e. protein pairs overlapping with the gold standard positives). FP is the number of false positives (i.e. protein pairs overlapping with the gold standard negatives).

The significance of a certain likelihood ratio value can be estimated using the hyper-geometric distribution:

Suppose that the total sample space is N. In the first round, a sample of size S_1 is randomly selected without replacement from N. Then, the entire initial sample (S_1) is subsequently returned to the sample space. In the second round, another sample of size S_2 is randomly selected without replacement from N. The size of the overlap (denoted as i) between the two samples (S_1 and S_2) is a hyper-geometric random variable. The probability of observing an overlap of a given size X or greater is calculated by the formula:

$$P(i \geq X) = 1 - \sum_{i=0}^{X-1} \frac{\binom{S_1}{i}\binom{N-S_1}{S_2-i}}{\binom{N}{S_2}}$$

To calculate the statistical significance of the overlap between the gold-standard positives and our predictions, we first need to determine the total sample space (N). Because there are about 6000 yeast proteins, the total number of possible interacting pairs is $1.8\text{x}10^7$ ($6000\text{x}6000/2 = 1.8\text{x}10^7$). Therefore, the sample space (N) is $1.8\text{x}10^7$; $S_1 = 8250$; $S_2 = 23295$; $X = 7889$. Please note that the same likelihood ratio may correspond to different significance values, because the likelihood ratio only depends on the ratio of TP/FP, but the P-value depends on the actual values of TP and FP.

All networks are significantly related

As discussed in the main text, we first used Pearson correlation coefficient to measure the association between different networks. The results show that most of the networks are, in fact, closely related. However, one problem with correlation coefficient is that it takes into account all pairs of proteins within the whole network. However, since the majority of pairs have a distance larger than the diameter, the coefficient actually measures the correlation between distant pairs. Supplementary Table 1A shows that all networks are significantly related (P values are all smaller than 10^{-4}), although the specific correlations are not very significant.

In total, we used seven different methods to measure the association between networks:

1. Cramer's V (V).

We divided all pairs of proteins in a network into three bins: 1) connected pairs; 2) close pairs (distance=2); 3) distant pairs (distance≥3). The χ^2 statistics were calculated on the 3x3 contingency table between any two networks.

The formula for Cramer's V is

$$V = \sqrt{\frac{\chi^2}{N \min(I-1, J-1)}}$$

where I and J are the numbers of rows and columns (in our case, $I = J = 3$), and N is the total number of gene pairs. Cramer's V lies between zero and one inclusive, equals zero when there is no association, and equals one when the association is perfect.

2. Pearson correlation coefficient (CC)

Given two random variables, X and Y, the Pearson correlation coefficient $CC(X; Y)$ is calculated by the formula:

$$CC(X;Y) = \frac{\sum XY - \frac{\sum X \sum Y}{N}}{\sqrt{(\sum X^2 - \frac{(\sum X)^2}{N})(\sum Y^2 - \frac{(\sum Y)^2}{N})}}$$

where X records the distances for all protein pairs in one network. Y records the distances for the corresponding pairs in another network. We only include protein pairs that show up in both networks for this calculation.

The Pearson correlation coefficient lies between -1 and 1.

3. Mutual information (I)

Given two random variables, X and Y, the Mutual Information $I(X; Y)$ between them measures how much information does one variable conveys about the other one. It is defined as the relative entropy (or Kullback-Leibler distance) between the joint distribution and the product distribution of X and Y, that is:

$$I(X;Y) = \sum_x \sum_y P(x,y) \log \frac{P(x,y)}{P(x)P(y)}$$

where $P(x, y)$ indicates the joint distribution of X and Y and $P(x)$ and $P(y)$ their marginal distributions. It is easy to prove that:

$$I(X; Y) = H(X) - H(X|Y) = H(Y) - H(Y|X) = I(Y;X)$$

where $H(X)$ and $H(Y)$ are the entropies of X and Y, and $H(X| Y)$ and $H(Y| X)$ are the conditional entropies of X given Y and Y given X respectively. This states that the information that Y conveys about X is the reduction in uncertainty about X due to the knowledge of Y (and vice-versa).

4. Contingency coefficient (C)

The formula for C is

$$C = \sqrt{\frac{\chi^2}{\chi^2 + N}}$$

It lies between zero and one. But it will never be equal to one.

5. Association score (A)

Two proteins are defined as "associated" if their distance is smaller than or equal to 2. Given two networks, the fraction of associated pairs in one network that are also associated in the other was calculated. Then, we also calculated the random expectation. The association score is just the ratio of the observed fraction over the random expectation.

6. Pearson correlation coefficient on binned data (Binned CC)

We calculated Pearson correlation coefficients between networks using the binned data that were produced when we calculated Cramer's *V*.

7. Mutual information on binned data (Binned I)

We calculated mutual information between networks using the binned data that were produced when we calculated Cramer's *V*.

All methods produce similar results as indicated by their correlations with Cramer's *V* used in the main text (see supplementary Table 1B).

Expectation of the essentiality of composite hubs

We use "h1" and "h2" to denote hubs in two networks and use "e" to denote essential genes. P(e) = 0.2 for there are about 1000 essential genes in yeast genome (~6000 genes). Because we define hubs as the top 20% of the nodes with the highest degrees, P(h1) = P(h2) = 0.2. Since we know that hubs tend to be essential genes, let us suppose P(e|h1) = P(e|h2) = 0.4 (the fraction of essential hubs in the interaction network is about 40%).

Then:
P(h1|e) = P(e|h1)P(h1)/P(e) = 0.4 * 0.2 / 0.2 = 0.4
P(h2|e) = P(e|h2)P(h2)/P(e) = 0.4 * 0.2 / 0.2 = 0.4
P(h1&h2|e) = P(h1|e)P(h2|e) = 0.4 * 0.4 = 0.16
P(h1|~e) = P(~e|h1)P(h1)/P(~e) = (1-0.4) * 0.2 / (1-0.2) = 0.15
P(h2|~e) = P(~e|h2)P(h2)/P(~e) = (1-0.4) * 0.2 / (1-0.2) = 0.15
P(h1&h2|~e) = P(h1|~e)P(h2|~e) = 0.15 * 0.15 = 0.0225
P(h1&h2) = P(h1&h2|e)P(e) + P(h1&h2|~e)P(~e) = 0.16*0.2+0.0225*(1-0.2) = 0.05
P(e|h1&h2) = P(h1&h2|e)P(e)/P(h1&h2) = 0.16 * 0.2 / 0.05 = 0.64

Therefore, P(e|h1&h2) > P(e|h1) = P(e|h2), i.e. composite hubs are more likely to be essential than normal hubs, simply because of the fact that normal hubs tend to be essential genes.

Expectation of the overlap of hubs between scale-free networks

Above, we have shown that P(h1&h2) = 0.05, given the fact that hubs in scale-free networks tend to be essential. If we assume that this were not the case, then

P'(h1&h2) = P(h1) * P(h2) = 0.2 * 0.2 = 0.04 < P(h1&h2).

Therefore, hubs in scale-free networks should tend to overlap just because of their essentiality.

Defining hubs with different cutoffs does not affect the results

In order to determine whether different definitions of hubs using different cutoffs will affect the final results, we repeated all calculations in Figures 2 and 3 using different cutoffs. As shown in SupFig. 3A, even though there is some fluctuation, the overlaps between the regulatory networks and action networks (particularly interaction and expression networks) are always very similar to random expectation (P values > 0.05), whereas, the overlaps between action networks are always significantly higher than random expectation (P values < 0.001) when the cutoff is smaller than 70%.

SupFig 3B clearly shows that hubs in different network have a strong tendency to be essential (except those in target population). Furthermore, tri-hubs and bi-hubs have even higher tendencies than normal hubs at all cutoffs. The fluctuation at the low cutoff end is due to the limited statistics, i.e., there are only few tri-hubs at those cutoffs.

Regulator bridges generate time-delayed expression relationships

Bridge motif:

$$\begin{array}{ccc} TF_1 & \longrightarrow & TF_2 \\ \downarrow & & \downarrow \\ T_1 & & T_2 \end{array}$$

We have constructed the following approximate chemical kinetic model for the bridge motif (see above) in the transcriptional regulatory network:

$$TF_1 + DNA_1 \underset{}{\overset{K_A}{\rightleftharpoons}} TF_1{\bullet}DNA_1$$

$$x\ NTP \xrightarrow[k_1]{TF_1 \bullet DNA_1} T_1^{mRNA} + x\ PPi$$

$$TF_1 + DNA_2 \underset{}{\overset{K_A}{\rightleftharpoons}} TF_1{\bullet}DNA_2$$

$$x\ NTP \xrightarrow[k_1]{TF_1 \bullet DNA_2} TF_2^{mRNA} + x\ PPi$$

$$y\ aa\text{-}tRNA \xrightarrow[k_2]{TF_2^{mRNA}} TF_2 + y\ tRNA$$

$$TF_2 + DNA_3 \underset{}{\overset{K_A}{\rightleftharpoons}} TF_2{\bullet}DNA_3$$

$$x\ NTP \xrightarrow[k_1]{TF_2 \bullet DNA_3} T_2^{mRNA} + x\ PPi$$

In this model, the first transcription factor TF_1 binds to the first DNA motif and activates the transcription of the first target T_1^{mRNA}. At the same time, TF_1 binds to a second DNA motif and activates the transcription of another transcription factor TF_2. After translation and translocation, the TF_2 protein then binds to a third DNA motif and activates the transcription of the second target T_2^{mRNA}. We assume that the binding between TF and the corresponding DNA motif is fast and reversible, with an association constant of K_A. k_1 is the rate constant for transcription, k_2 is the combined rate constant for translation and translocation. We assume that K_A and k_1 are the same for both transcription factors. NTP, PPi, and aa-tRNA represent nucleoside triphosphate, pyrophosphate, and aminoacyl-tRNA, respectively.

The above model corresponds to the following set of equations:

$$\frac{[TF_1 \bullet DNA_1]}{[TF_1][DNA_1]} = \frac{[TF_1 \bullet DNA_2]}{[TF_1][DNA_2]} = \frac{[TF_2 \bullet DNA_3]}{[TF_2][DNA_3]} = K_A$$

$$[TF_1 \bullet DNA_1] + [DNA_1] = [TF_1 \bullet DNA_2] + [DNA_2] = [TF_2 \bullet DNA_3] + [DNA_3] = c_D$$

$$\frac{d}{dt}[T_1^{mRNA}] = k_1[TF_1 \bullet DNA_1]$$

$$\frac{d}{dt}\left([TF_2^{mRNA}] + [TF_2] + [TF_2 \bullet DNA_3]\right) = k_1[TF_1 \bullet DNA_2]$$

$$\frac{d}{dt}\left([TF_2] + [TF_2 \bullet DNA_3]\right) = k_2[TF_2^{mRNA}]$$

$$\frac{d}{dt}[T_2^{mRNA}] = k_1[TF_2 \bullet DNA_3]$$

Here we use [A] to denote the concentration of molecular species A. We assume that there is one copy of each DNA motif in the genome, so that the concentration for each DNA motif is the same as the concentration of DNA molecules in the nucleus, c_D. We also assume that the concentrations of NTPs are roughly constant, so that the rate of transcription only depends on TF binding to DNA motif. Similarly, we assume that the concentrations of aa-tRNAs are roughly constant, so that the rate of translation only depends on the concentration of mRNA. Furthermore, we initialize the parameters in the following way:

$$[TF_1] = \begin{cases} 1000c_D, & t \geq 0 \\ 0, & t < 0 \end{cases}$$

$$K_A = 0.01 / c_D$$

$$k_2 = 0.1k_1$$

These parameters can be understood in the following intuitive way. When $t<0$, TF_1 is absent in the nucleus. When $t \geq 0$, the number of TF_1 molecules in the nucleus rises to a constant of 1000 per DNA molecule. The association constant between TF and the

corresponding DNA motif is defined such that there need to be at least 100 free TF molecules per DNA molecule to ensure that the DNA motif is bound to TF half the time. Furthermore, we assume that the combined rate constant of translation and translocation is ten times slower than the rate constant of transcription.

We numerically simulate the time evolution of the concentration of two target mRNAs: T_1^{mRNA}, and T_2^{mRNA} (Supplementary Fig.9). It is clear from the simulation that the two targets have a time-delayed expression relationship.

Our model can be improved by incorporating additional processes such as mRNA and protein degradation, and by considering stochastic gene expression. However, the conclusion that the two targets have a time-delayed expression relationship will still hold.

Disconnected pairs in the metabolic network tend to have expression relationships other than co-expression

We obtained expression profiles of yeast genes through two complete cell cycles [6]. Between the expression profiles of pairs of genes, we used a local clustering method to calculate four types of temporal relationships: correlated (i.e. co-expressed), time-shifted, inverted, and inverted time-shifted [45]. To find these relationships, expression levels must be assessed over a time-course, with many measurements, at small and uniform intervals. Most available datasets do not satisfy these conditions, being only suitable for simple correlation calculations (*i.e.,* co-expression); thus, we can only conduct detailed analysis on the cell-cycle datasets [6, 34].

We examined the occurrences of co-expression and shifted motifs using three cell-cycle datasets [34]: (1) Alpha (2)Cdc-15 (3) Elutriation. Supplementary Figure 10 shows that the results in all three datasets are very similar to those in the main text, i.e. disconnected pairs in the metabolic network tend to have expression relationships other than co-expression. However, similar results could not be found in the interaction dataset (see supplementary Figure 11).

Defining expression networks with different cutoffs does not affect the results

In order to determine whether different definitions of expression networks using different cutoffs will affect the final results, we repeated all calculations in Figures 3, 4 and 5 using different cutoffs. Supplementary Figures 12 and 13 clearly shows that all results remain the same.

P values by cumulative binomial distribution

Most P values in our analysis measure whether the difference is significant between the testing and control groups. They are calculated using the cumulative binomial distribution:

$$P(c \geq c_o) = \sum_{c=c_o}^{N} [\frac{N!}{N!(N-c)!}] p^c (1-p)^{N-c}$$

where N is the total number of possible gene pairs in the data; c_o is the number of observed pairs with a specific relationship in the testing group; and p is the probability of finding a gene pair with the same relationship in the control group. In this manner, we are testing whether gene pairs with a specific relationship are over-represented as compared to the control group. If they are under-represented, then $P(c < c_o) = 1 - P(c \geq c_o)$.

Tables

Supplementary Table 1.

A. P values for Pearson correlation coefficients

	CC	P values
**Met-Int**	1.25E-01	2.20E-16
**Met-Reg**	1.70E-02	1.60E-05
**Met-Exp**	2.50E-02	1.80E-15
**Reg-Int**	5.50E-02	2.20E-16
**Reg-Exp**	5.50E-02	2.20E-16
**Int-Exp**	4.20E-02	2.20E-16

B. Correlation coefficients between *V* and other measurements

	V	CC	I	C	A	Binned CC	Binned I
**Met-Int**	8.57E-02	1.25E-01	4.87E-02	1.46E-01	2.70E+00	2.96E-01	4.06E-02
**Met-Reg**	4.13E-03	1.70E-02	9.00E-03	8.20E-03	1.62E+00	8.68E-02	4.73E-03
**Met-Exp**	2.44E-03	2.50E-02	6.58E-03	4.85E-03	1.45E+00	6.56E-02	2.67E-03
**Reg-Int**	3.52E-03	5.50E-02	5.08E-03	6.99E-03	1.71E+00	5.29E-02	2.60E-03
**Reg-Exp**	2.59E-03	5.50E-02	4.40E-03	5.16E-03	1.75E+00	6.18E-02	2.53E-03
**Int-Exp**	6.33E-03	4.20E-02	3.71E-03	1.25E-02	1.78E+00	5.72E-02	2.13E-03
**correlation**	1.00E+00	9.14E-01	9.92E-01	1.00E+00	9.57E-01	9.91E-01	9.97E-01

Supplementary Table 2. TFs regulating the same genes tend to interact and co-express with each other

A. Likelihood ratios for TFs in the same motif to interact

Motifs	# of pairs	# of TPs	# of FPs	Likelihood ratios
**All**	4672	100	297	39.15
**MIM**	601	14	8	203.50
**FFL**	246	13	26	58.14

B. Likelihood ratios for TFs in the same motif to co-express

Motifs	# of pairs	# of TPs	# of FPs	Likelihood ratios
**All**	4534	17	918	2.54
**MIM**	601	5	110	6.24
**FFL**	240	2	39	7.03

Supplementary Table 3. Bridges are enriched in most disconnected enzymes with different distances in the metabolic network

**Distance**	1	2	3	4	5	6	7	8	9
**P-values#**	0.1	0	5.48E-11	1.89E-06	0	0	1.92E-13	2.13E-12	0.03
**Distance**	10	11	12	13	14	15	16	17	Overall*
**P-values#**	0.05	0.002	0.38	0.005	0.02	0.14	0.23	0.05	0

#: P-values measure the significance of the difference between the observed fraction of bridges and the random expectation at different distances.
*: The P-value for overall measures the significance of the difference between the observed fraction of bridges and the random expectation for all disconnected enzyme pairs.

Figure captions

Supplementary Figure 1. Essentiality of bi-hubs. We created randomized networks where the tendency for hubs to be essential is conserved, and compared observed essentiality enrichment in bi-hubs with calculations based on the randomized networks. All other calculations are the same as in Figure 2B.

Supplementary Figure 2. Fold enrichments of hub overlaps (O) between two networks relative to random expectation. We created randomized networks where the tendency for hubs to be essential is conserved, and compared observed essentiality enrichment in composite-hubs with calculations based on the randomized networks. All other calculations are the same as in Figure 3B.

Supplementary Figure 3. We recalculated Figure 3B and Figure2 using different percentile cutoffs for defining hubs. Generally speaking, results are not affected by the specific definition of hubs. Some of the results at extremely low percentiles (< 10%) are not very stable, because there are few hubs defined under these percentile cutoffs.

Supplementary Figure 4. Functional composition of different networks and their overlap. Clearly, all four networks have similar functional compositions, except that the regulatory network is enriched in class 4 proteins (Class 4: Transcription) and the metabolic network is enriched in class 1 proteins (Class 1: Metabolism). Both exceptions are readily understandable based on the nature of the networks. Furthermore, the functional composition of the overlapping proteins between the four networks is really similar to the random expectation. The P value measures the statistical significance of the difference between a certain distribution with the random expectation using a K-S test. All P values are bigger than 0.05, confirming that all functional compositions are similar to the random expectation.

Supplementary Figure 5. Changes of L in the four networks when hubs in one of them are removed. (A). Hubs in the metabolic network are removed. It is clear that, as more and more hubs in the metabolic network are removed, the L's of interaction and expression networks go down very quickly (i.e., the networks begin to collapse), as compared to that of the regulatory network, which remains around 6.3. (B) Hubs in the regulatory network are removed. As more and more hubs are removed, the L of the regulatory network goes down very quickly, whereas the L's of all three action networks stay stable. These results confirms our conclusion that separation of hubs between regulatory and action networks provides stability to the cell.

Supplementary Figure 6A. Examples of triangles. In all panels of supplementary Figures 6 and 7, circles represent TFs, and rectangles represent non-TF genes. (I) A schematic of a triangle. The schematic shows that a triangle consists of three proteins: the common regulator TF regulates both P1 and P2. In our calculations, we examined the occurrences of triangles between not only adjacent pairs in action networks, but also distant pairs (distance = k). In all panels of supplementary Figures 6 and 7, P1 and P2

have a distance of *k* in a certain action network. In all examples in supplementary Figures 6&7, *k* is equal to one. (II) A triangle in Met-Reg. *ADE5, 7* and *ADE8* are two enzymes in the purine biosynthesis pathway. *ADE5,7* turns 5-phosphoribosylamine into 5'-phosphoribosylglycinamide (GAR), which is subsequently turned into 5'-phosphoribosyl-N-formylglycinamide by *ADE8*. These two enzymes are co-regulated by *BAS1*. (III) A triangle in Int-Reg. *SCC1* and *SCC3* are two subunits of the cohesin complex. They are co-regulated by *MBP1*. In supplementary Figures 6A(III), B(III) and C(III), the dotted lines indicate the interaction relationship, and the dotted rectangles represent interaction partners of the two proteins. (IV) A triangle in Exp-Reg. *MET10* and *MET14* are two enzymes involved in methionine metabolism. The Pearson correlation coefficient between their expression profiles is 0.84. They are co-regulated by *MET28*. In supplementary Figures 6A(IV), B(IV) and C(IV), the dotted lines indicate the co-expression relationship, and the dotted rectangles represent genes that are co-expressed with the two genes.

Supplementary Figure 6B. Examples of trusses. (I) A schematic of a truss. The schematic shows that a truss consists of four proteins: T1 regulates T2, P1 and P2; T2 regulates P1 and P2. (II) A truss in Met-Reg. *GLT1* and *GLN1* are two enzymes involved in glutamate metabolism. *GLT1* turns 2-oxoglutarate into L-glutamate, which is subsequently turned into L-glutamine by *GLN1*. These two enzymes are co-regulated by *GLN3* and *GCN4*. *GLN3* also regulates *GCN4*. (III) A truss in Int-Reg. *CLN1* and *CLN2* are two subunits of the *CDC28*-associated complex. They are co-regulated by *MBP1* and *SWI4*. *MBP1* also regulates *SWI4*. (IV) A truss in Exp-Reg. The expression of *HSP78* and *HSP82* are both induced in heat-shock response. The Pearson correlation coefficient between their expression profiles is 0.86. They are co-regulated by *SKN7* and *YAP1*. *SKN7* also regulates *YAP1*.

Supplementary Figure 6C. Examples of bridges. (I) A schematic of a bridge. The schematic shows that a bridge consists of four proteins: T1 regulates T2 and P1; T2 regulates P2. (II) A bridge in Met-Reg. *FOL2* and *PHO8* are two enzymes involved in the folate biosynthesis pathway. *FOL2* turns 2,5-diamino-6-(5'-triphosphoryl-3',4'-trihydroxy-2'-oxopentyl)- amino-4-oxopyrimidine into 6-(l-erythro-1,2-Dihydroxypropyl 3-triphosphate)-7,8-dihydropterin, which is subsequently turned into dihydroneopterin by *PHO8*. *FOL2* is regulated by *YOX1*. *PHO8* is regulated by *PHO4*. *YOX1* also regulates *PHO4*. (III) A bridge in Int-Reg. *SPC2* and *SEC11* are two subunits of the signal peptidase complex. *SPC2* and *SEC11* are regulated by *ROX1* and *YAP6*, respectively. *ROX1* also regulates *YAP6*. (IV) A bridge in Exp-Reg. *MET10* and *MET20* are two enzymes involved in methionine metabolism. The Pearson correlation coefficient between their expression profiles is 0.87. *MET10* is regulated by *CBF1*. *MET20* is regulated by *REB1*. *CBF1* also regulates *REB1*.

Supplementary Figure 7. Examples of composite motifs in the combined network of Met-Exp. (A) A schematic of composite motifs in Met-Exp. The schematic shows that composite motifs in Met-Exp consist of two proteins: P1 and P2. P1 and P2 have a distance of *k* in the metabolic network. They also have an expression relationship (co-expressed or others) in the expression network. (B) A co-expression motif in Met-Exp.

ACS2 and *ERG10* are two enzymes involved in pyruvate metabolism. *ACS2* turns acetyladenylate into acetyl-CoA, which is subsequently turned into acetoacetyl-CoA by *ERG10*. *ACS2* and *ERG10* have a co-expression relationship (the local clustering score is 14.6). (C) A shifted motif in Met-Exp. *HEM13* and *HEM14* are two enzymes involved in porphyrin and chlorophyll metabolism. *HEM13* turns coproporphyrinogen III into protoporphyrinogen IX, which is subsequently turned into protoporphyrin IX by *ERG10*. *HEM13* and *HEM14* have a time-shifted relationship (the local clustering score is 14.9). |→| indicates a time-shift relationship.

Supplementary Figure 8. Fraction (F) of all P1-P2 pairs at distance k=1 in a given highly combined network in a particular composite motif.

Supplementary Figure 9. Simulated time evolution of the concentration of two target mRNAs: (A) T_1^{mRNA}, and (B) T_2^{mRNA}. X-axis represents time multiplied by the rate constant k_1. Y-axis represents the concentration of each molecular species divided by c_D. Both axes represent dimensionless quantities.

Supplementary Figure 10. Fraction (F) of all P1-P2 pairs at distance k in Met-Exp in a particular composite motif using three different cell-cycle datasets: (1) Alpha (2)Cdc-15 (3) Elutriation.

Supplementary Figure 11. Fraction (F) of all P1-P2 pairs at distance k in Int-Exp in a particular composite motif.

Supplementary Figure 12. Fold enrichments of hub overlaps (O) between two networks relative to random expectation. The expression networks in different panels are defined by different cutoffs. Exp5: the cutoff is 0.5; Exp6: the cutoff is 0.6; Exp7: the cutoff is 0.7; Exp85: the cutoff is 0.85.

Supplementary Figure 13. Fraction (F) of all P1-P2 pairs at distance k in a given combined network in a particular composite motif.

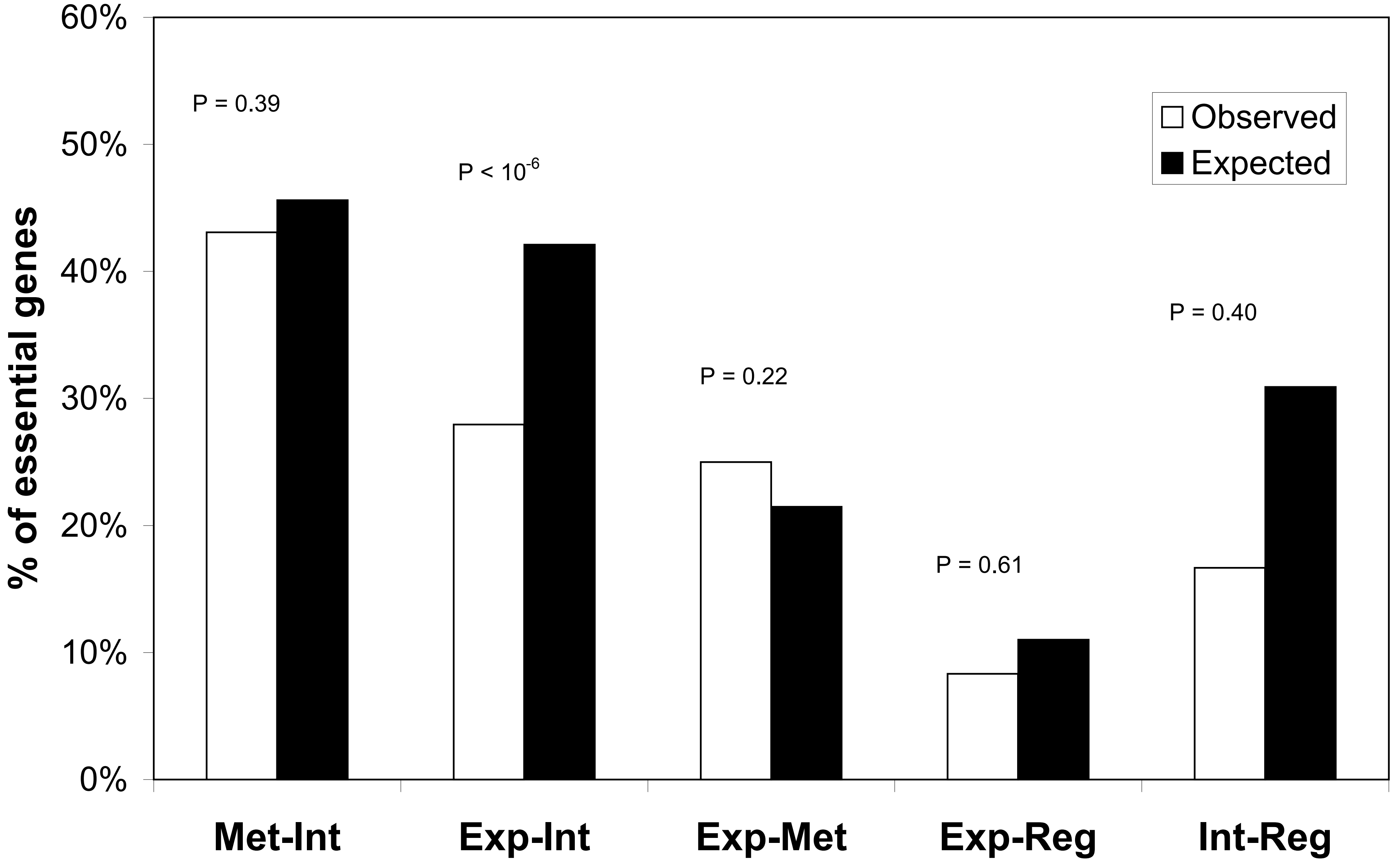

60%
50%
40%
30%
20%
10%
0%
% of essential genes
P = 0.39
P < 10-6
P = 0.22
P = 0.61
P = 0.40
Observed
Expected
Met-Int
Exp-Int
Exp-Met
Exp-Reg
Int-Reg

SupFig 1

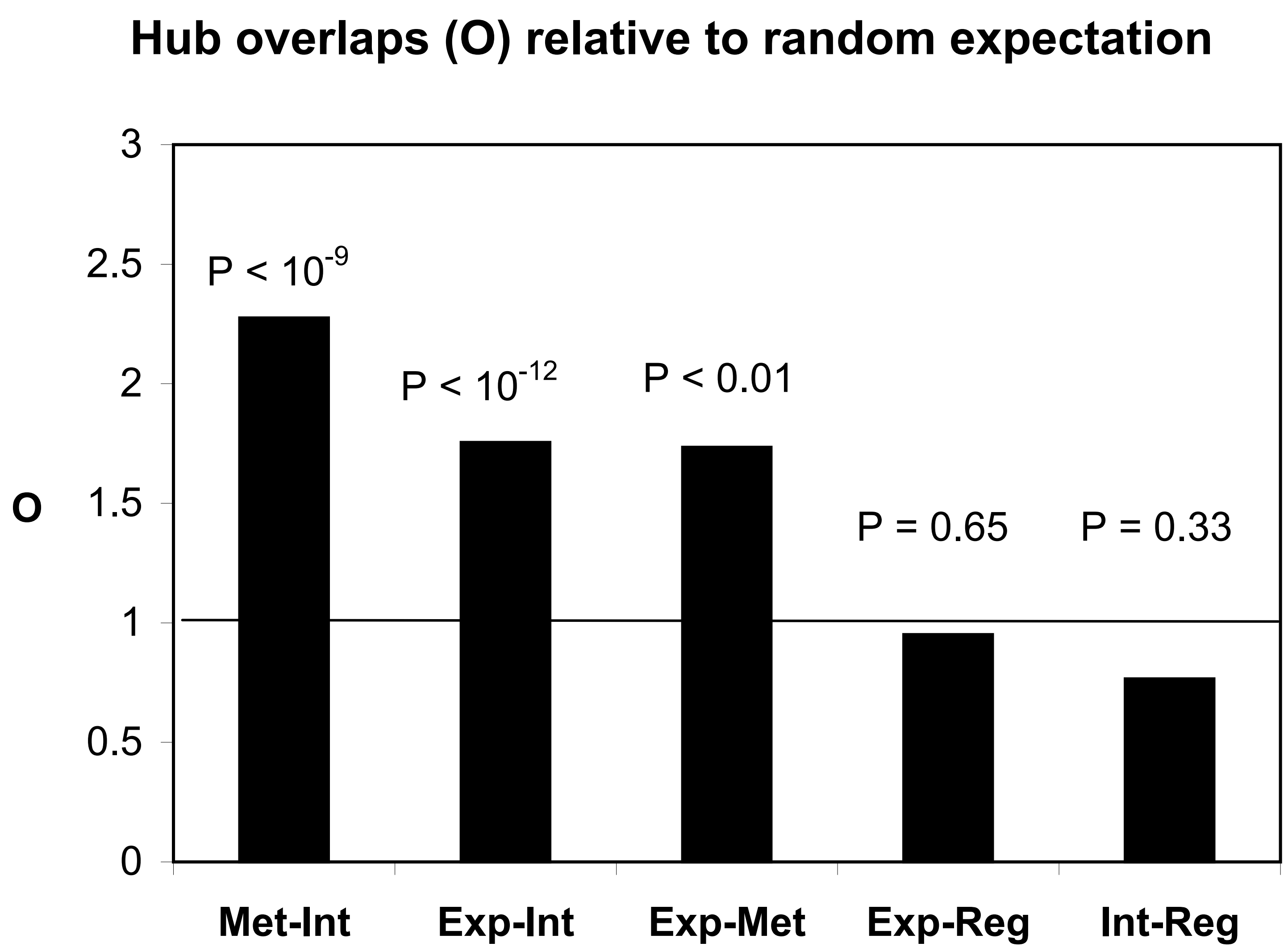
Hub overlaps (O) relative to random expectation
O
3
2.5
2
1.5
1
0.5
0
P < 10-9
P < 10-12
P < 0.01
P = 0.65
P = 0.33
Met-Int
Exp-Int
Exp-Met
Exp-Reg
Int-Reg

SupFig 2

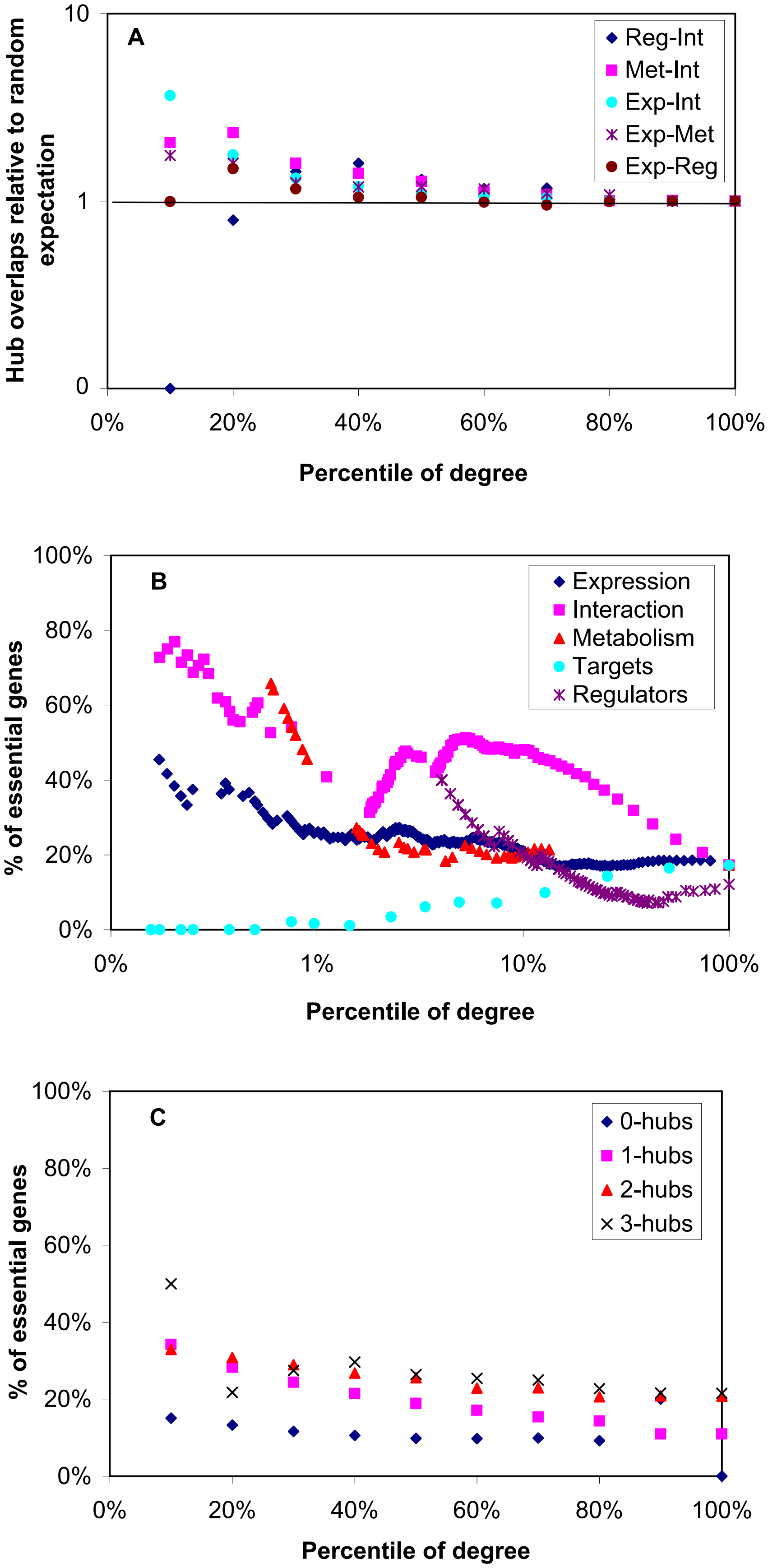
A
Reg-Int
Met-Int
Exp-Int
Exp-Met
Exp-Reg
Hub overlaps relative to random expectation
10
1
0
0%
20%
40%
60%
80%
100%
Percentile of degree
B
Expression
Interaction
Metabolism
Targets
Regulators
% of essential genes
100%
80%
60%
40%
20%
0%
0%
1%
10%
100%
Percentile of degree
C
0-hubs
1-hubs
2-hubs
3-hubs
% of essential genes
100%
80%
60%
40%
20%
0%
0%
20%
40%
60%
80%
100%
Percentile of degree

SupFig 3

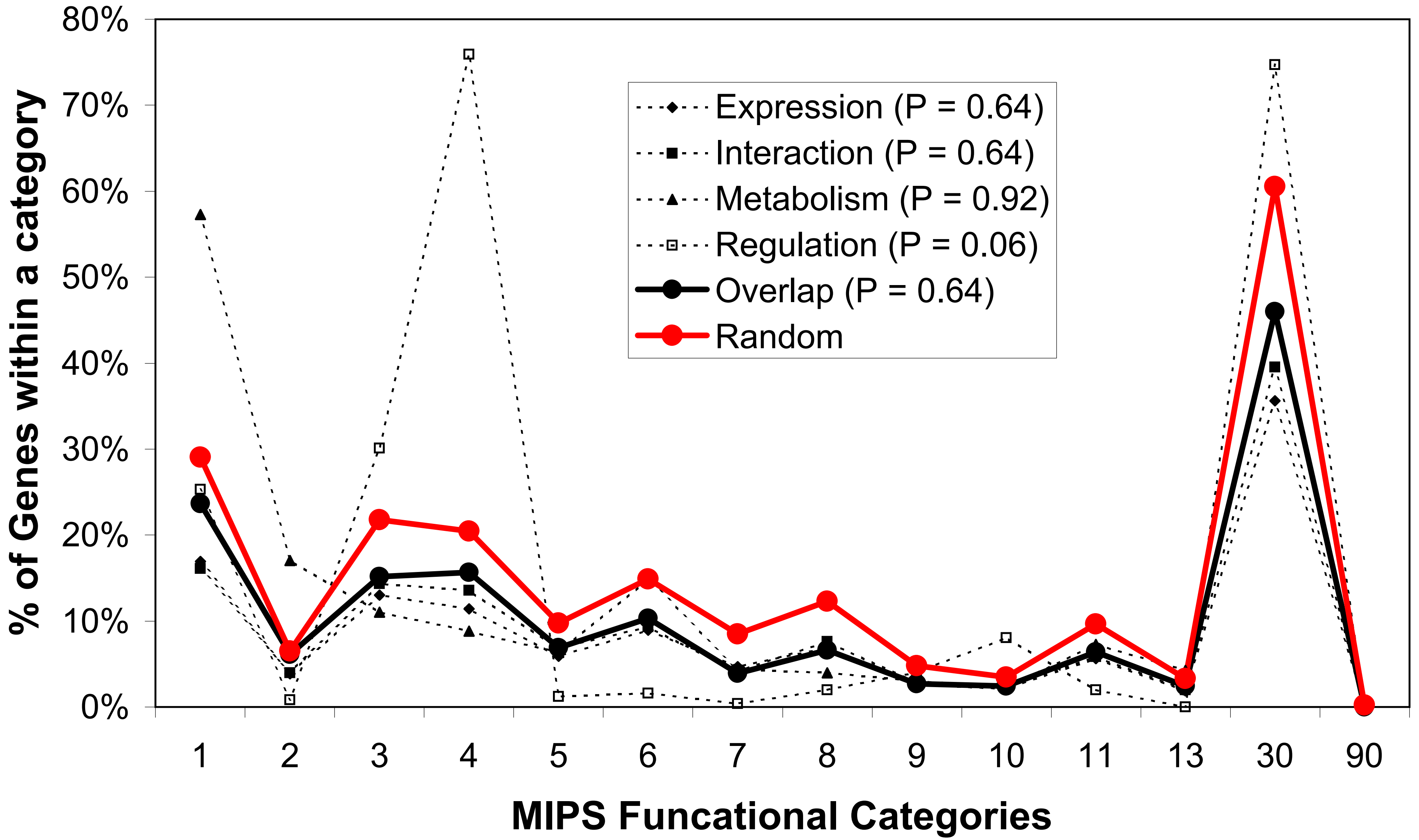
80%
70%
60%
50%
40%
30%
20%
10%
0%
% of Genes within a category
Expression (P = 0.64)
Interaction (P = 0.64)
Metabolism (P = 0.92)
Regulation (P = 0.06)
Overlap (P = 0.64)
Random
1
2
3
4
5
6
7
8
9
10
11
13
30
90
MIPS Funcational Categories

SupFig 4

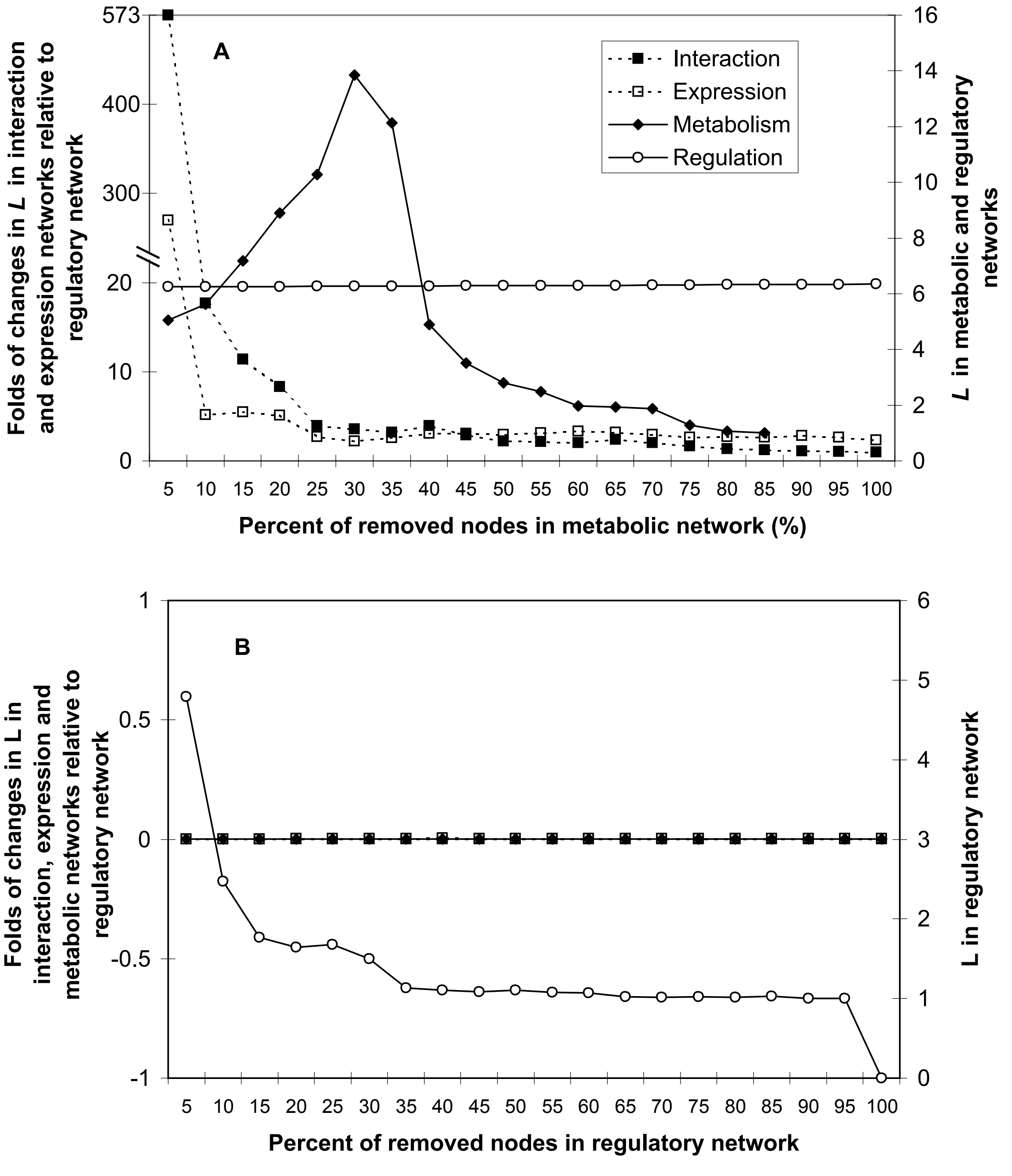
A
Interaction
Expression
Metabolism
Regulation
Folds of changes in L in interaction and expression networks relative to regulatory network
L in metabolic and regulatory networks
Percent of removed nodes in metabolic network (%)
573
400
300
20
10
0
16
14
12
10
8
6
4
2
0
5 10 15 20 25 30 35 40 45 50 55 60 65 70 75 80 85 90 95 100
B
Folds of changes in L in interaction, expression and metabolic networks relative to regulatory network
L in regulatory network
Percent of removed nodes in regulatory network
1
0.5
0
-0.5
-1
6
5
4
3
2
1
0
5 10 15 20 25 30 35 40 45 50 55 60 65 70 75 80 85 90 95 100

SupFig 5

A. Triangles

I. Schematic of a triangle

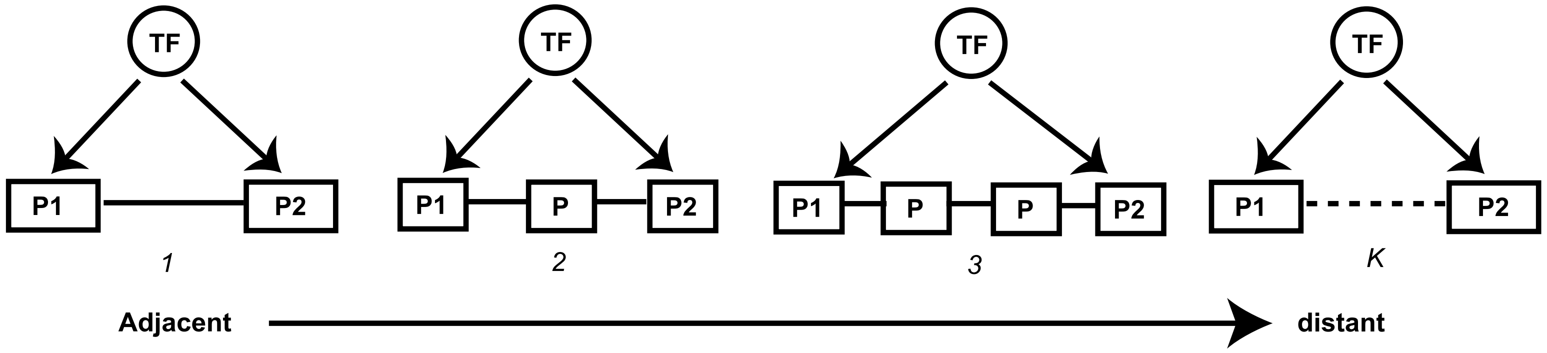

II. Metabolic network: Purine Biosynthesis

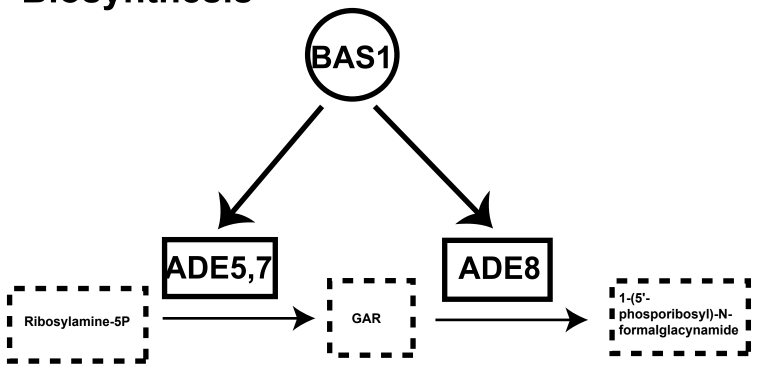

III. Interaction network

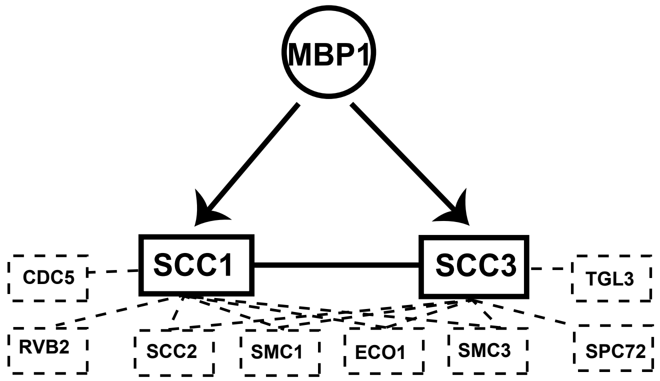

IV. Expression network

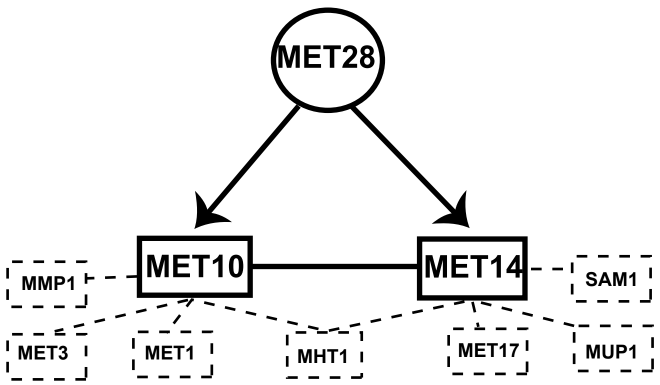

SupFig 6A

B. Trusses

I. Schematic of a truss

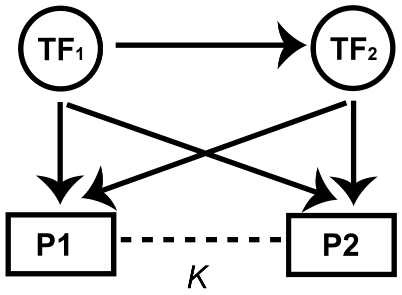

II. Metabolic network: Glutamate metabolism

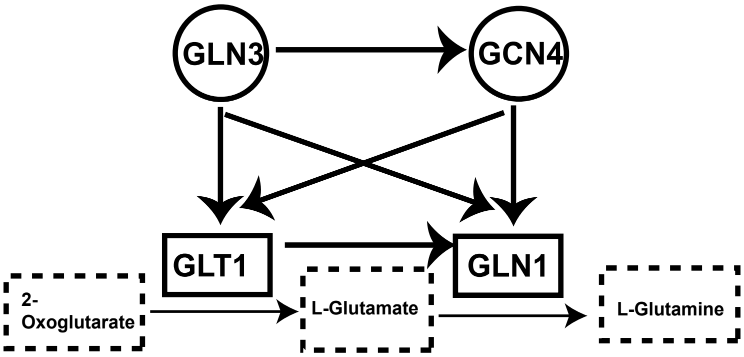

III. Interaction network

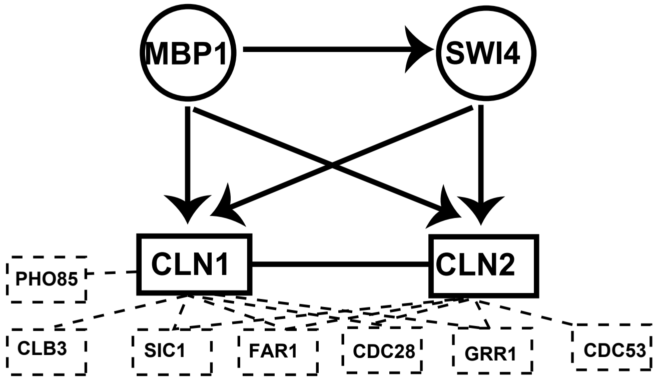

IV. Expression network

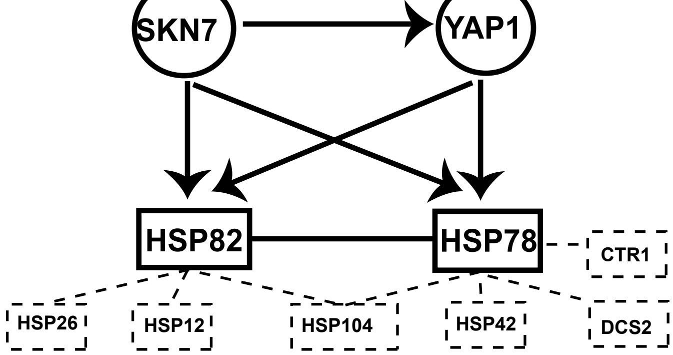

SupFig 6B

C. Bridges

I. Schematic of a bridge

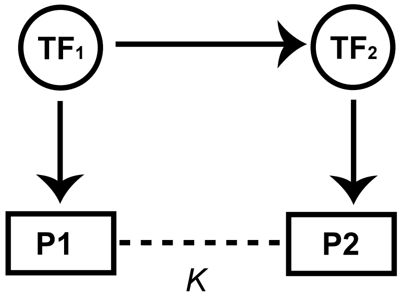

II. Metabolic network: Folate Biosynthesis

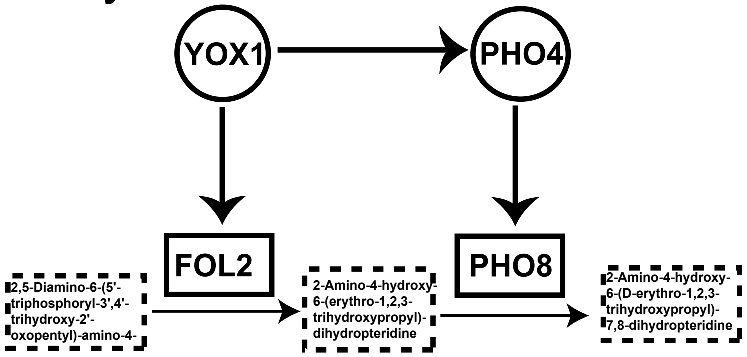

III. Interaction network

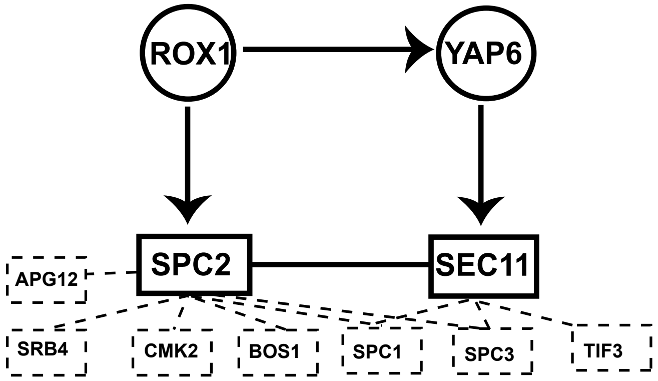

IV. Expression network

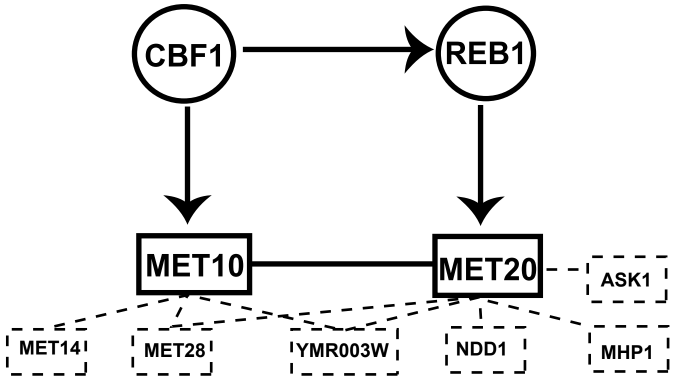

SupFig 6C

A. Schematic of composite motifs in Met-Exp

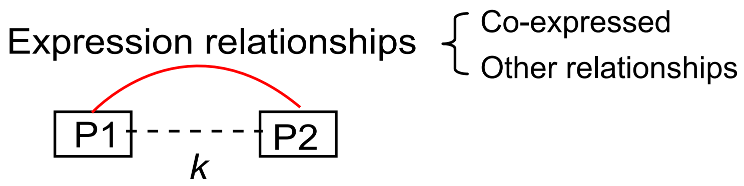

B. Co-expression motif

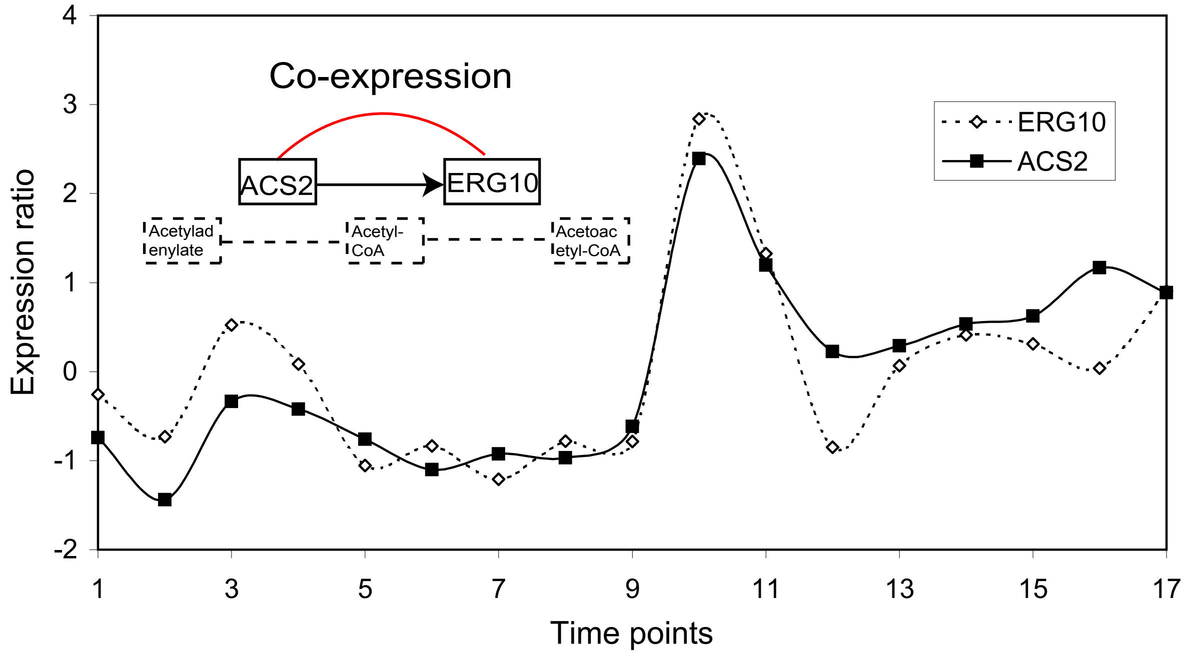

C. Shifted motif

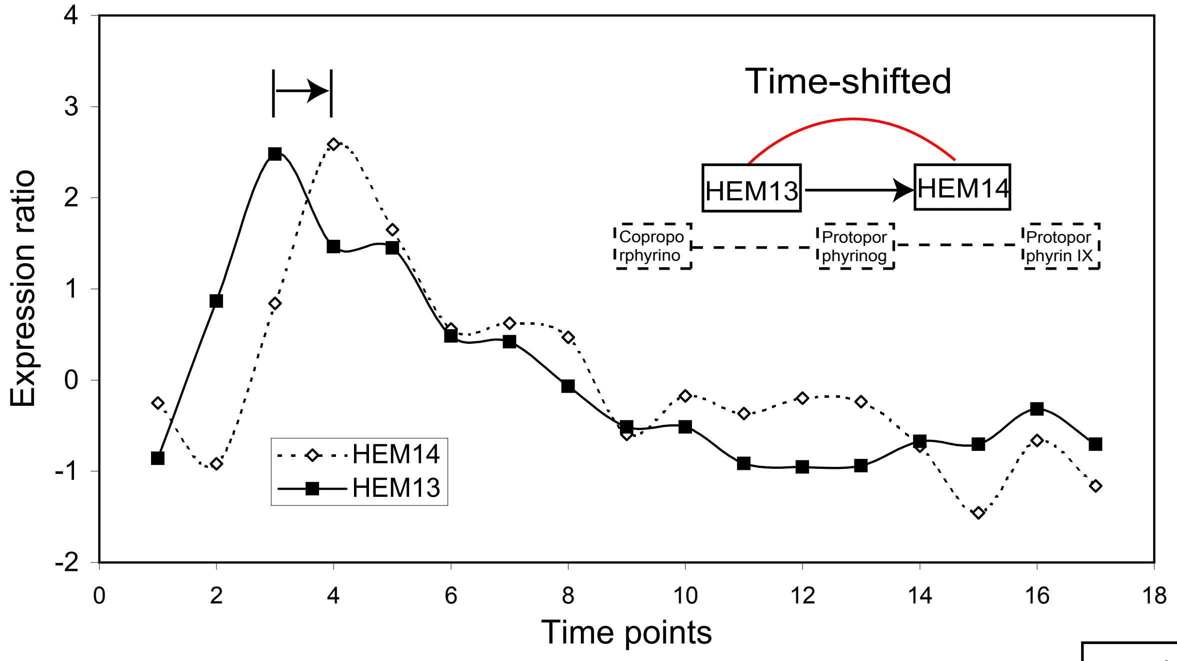

SupFig 7

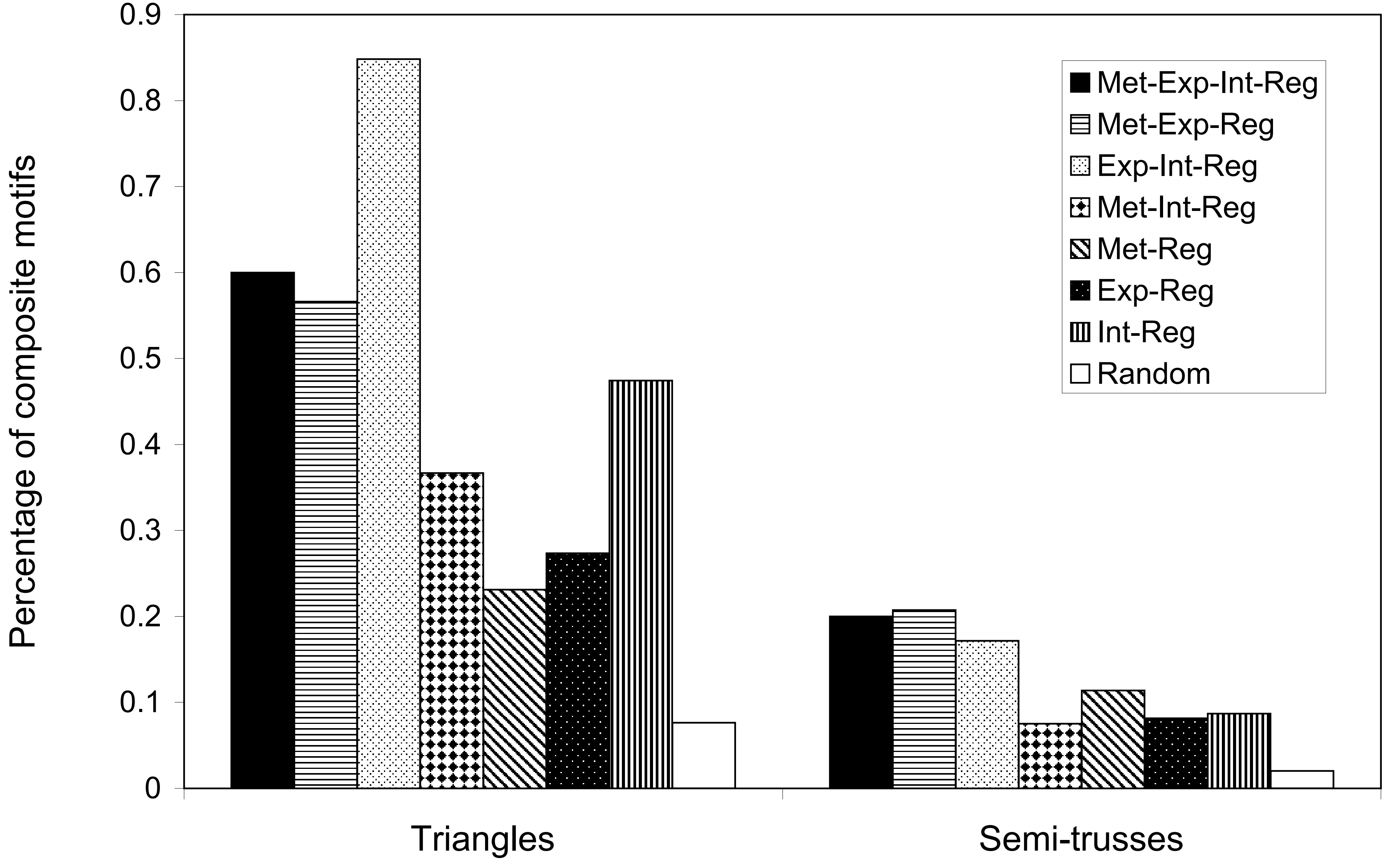

Percentage of composite motifs
0.9
0.8
0.7
0.6
0.5
0.4
0.3
0.2
0.1
0
Triangles
Semi-trusses
Met-Exp-Int-Reg
Met-Exp-Reg
Exp-Int-Reg
Met-Int-Reg
Met-Reg
Exp-Reg
Int-Reg
Random

SupFig 8

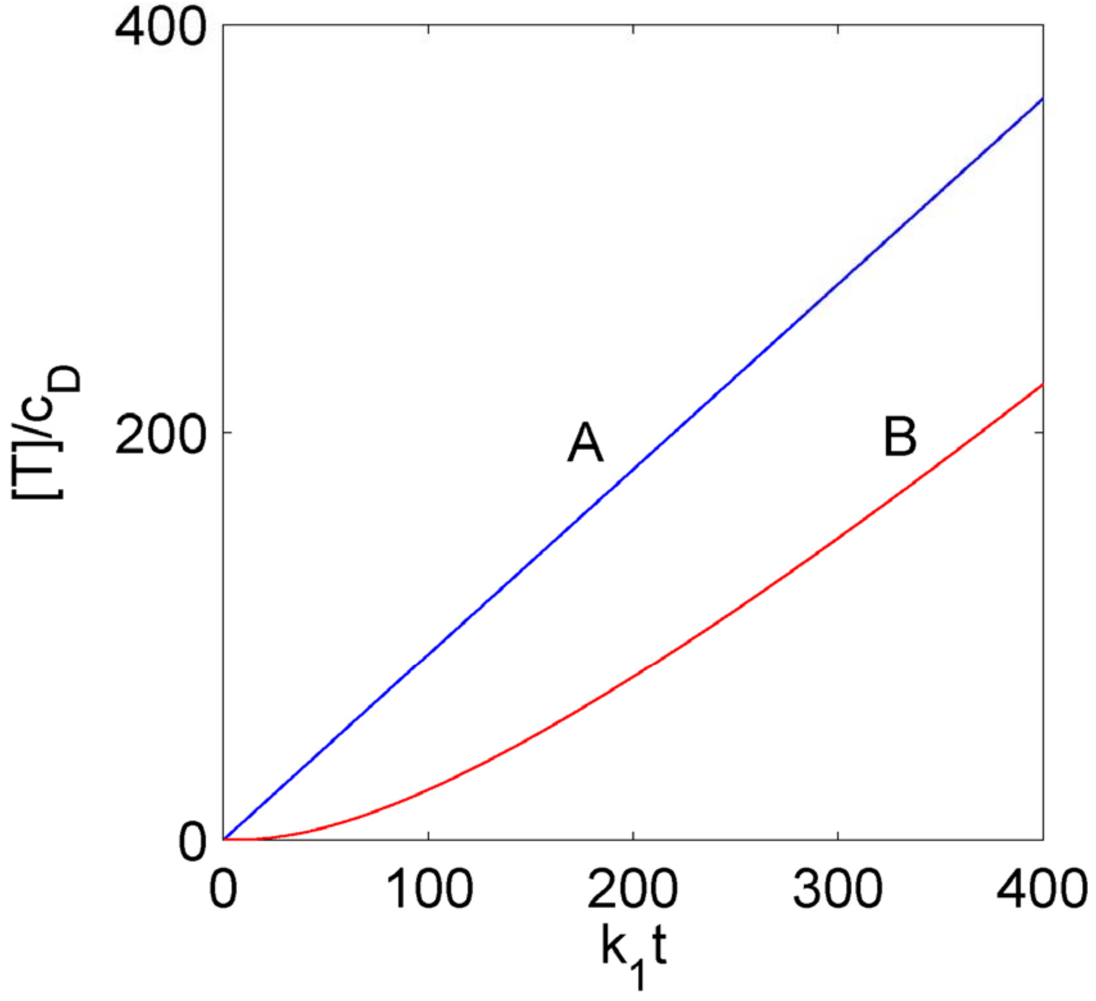

SupFig 9

Fraction (F) of all P1-P2 pairs at distance k in a given combined network in a particular composite motif

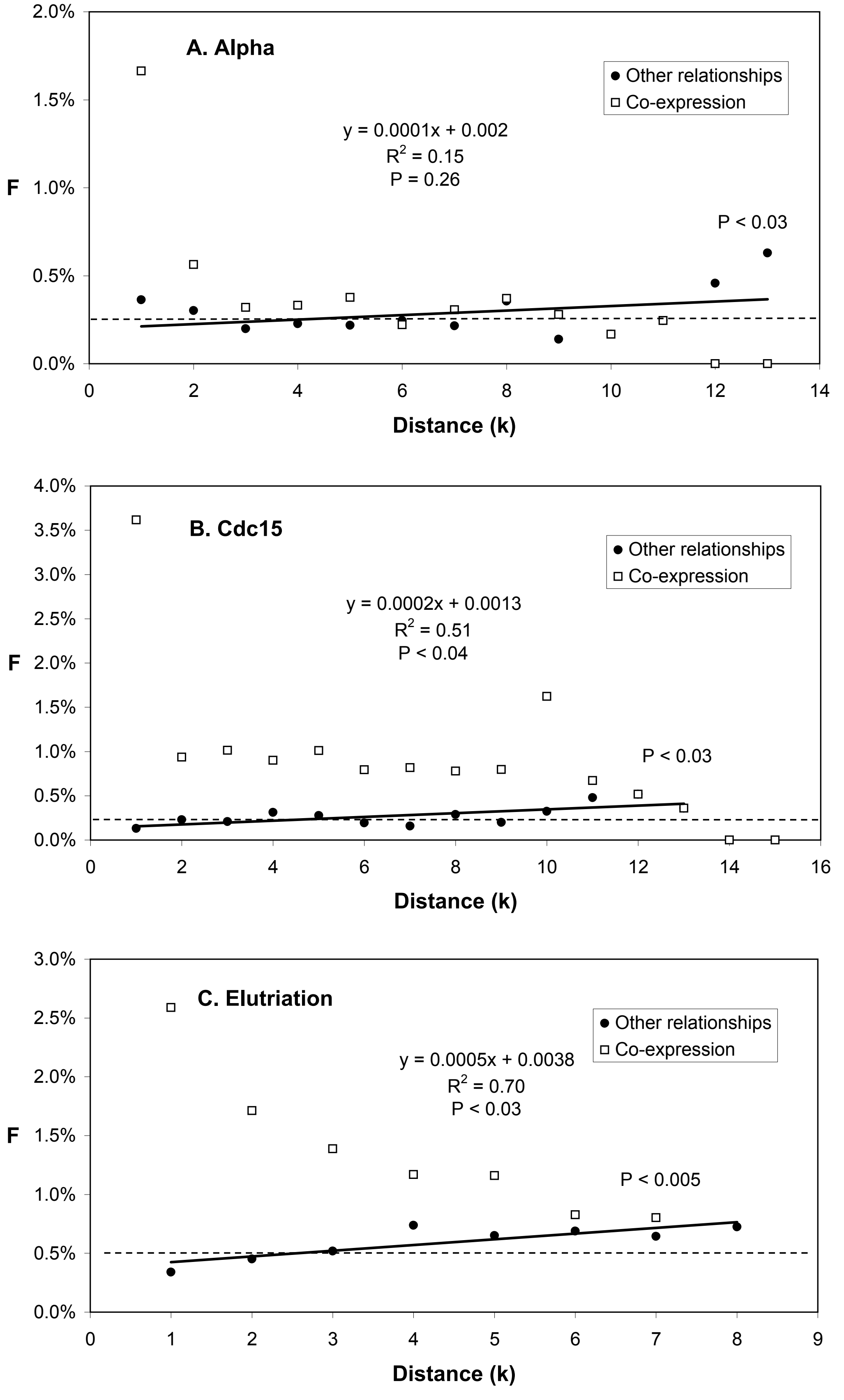

SupFig 10

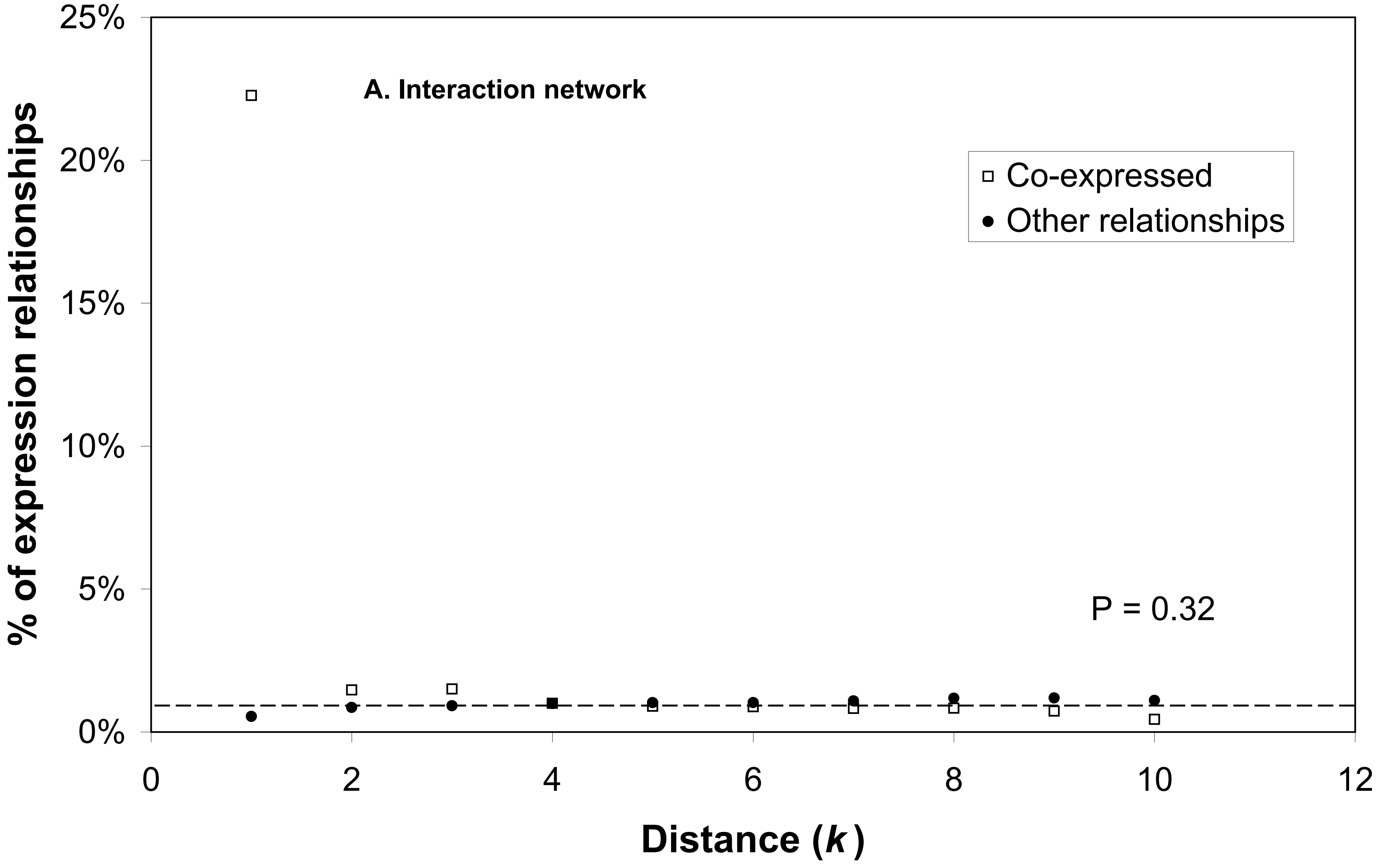

A. Interaction network
Co-expressed
Other relationships
P = 0.32
% of expression relationships
0%
5%
10%
15%
20%
25%
0
2
4
6
8
10
12
Distance (*k*)

SupFig 11

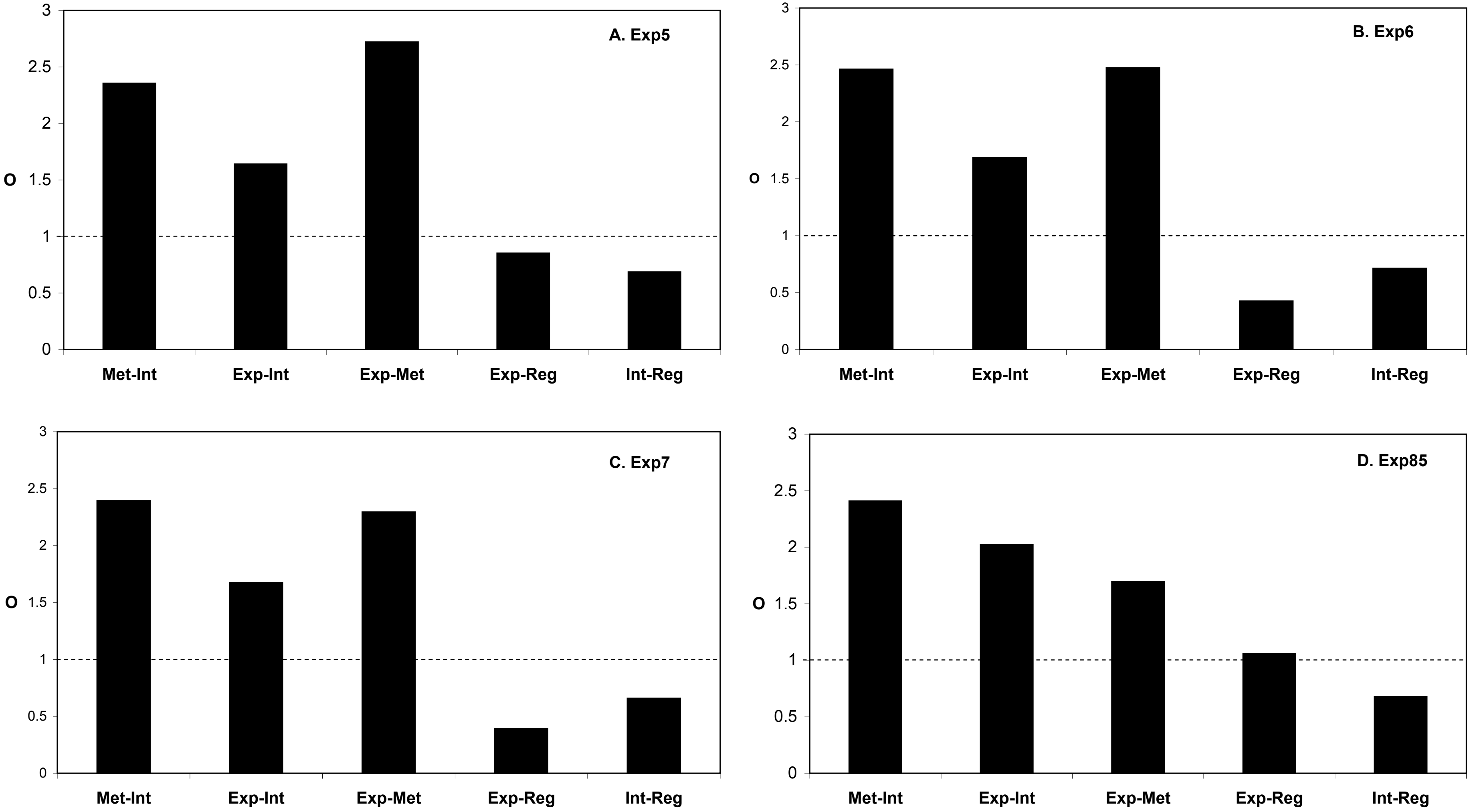
A. Exp5
B. Exp6
C. Exp7
D. Exp85
O
0
0.5
1
1.5
2
2.5
3
Met-Int
Exp-Int
Exp-Met
Exp-Reg
Int-Reg

SupFig 12

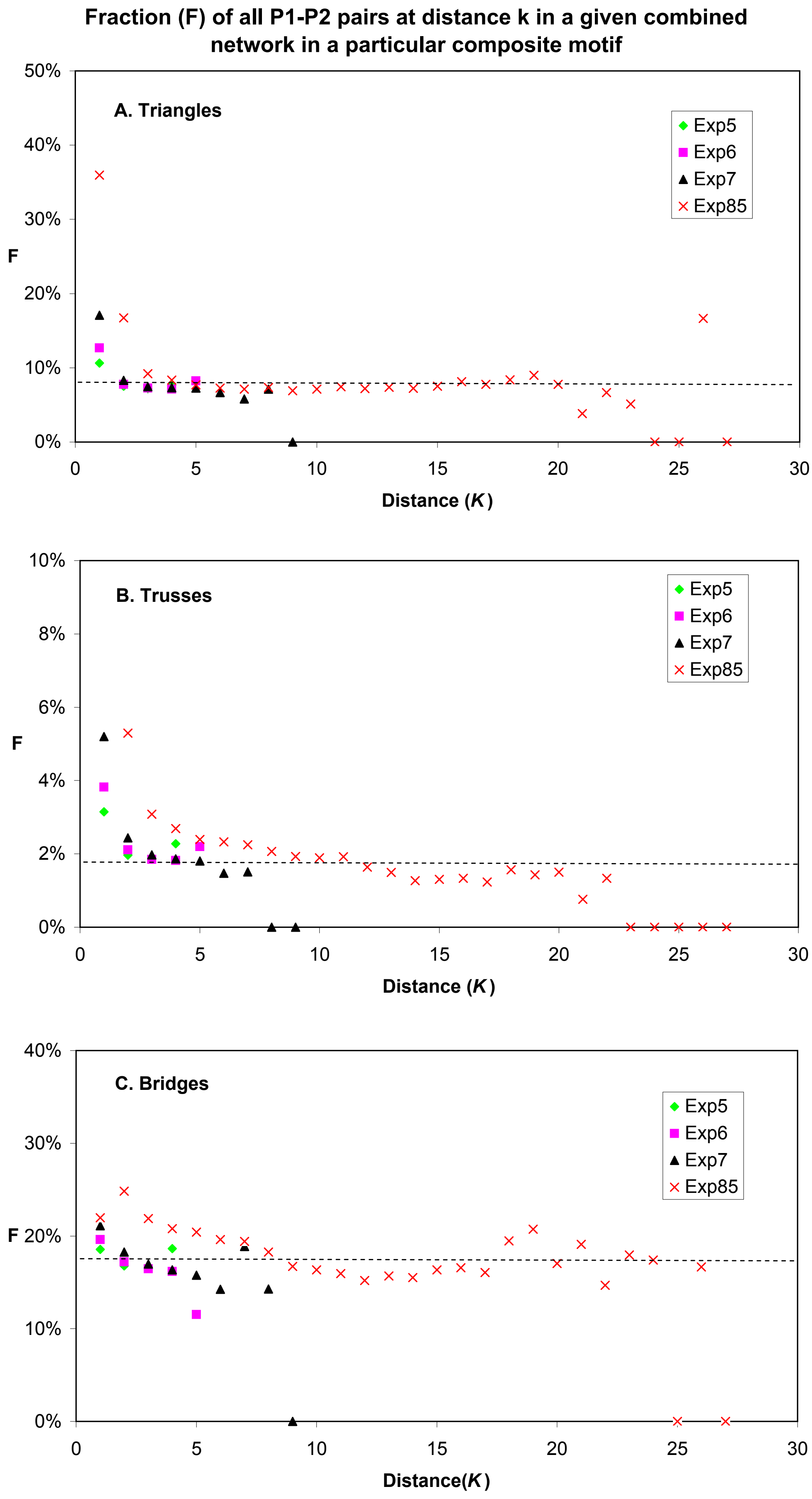
Fraction (F) of all P1-P2 pairs at distance k in a given combined network in a particular composite motif
A. Triangles
B. Trusses
C. Bridges
Exp5
Exp6
Exp7
Exp85
F
Distance (K)
0%
10%
20%
30%
40%
50%
2%
4%
6%
8%
0
5
10
15
20
25
30

SupFig 13
